# Hollow Metal–Organic Framework/MXene/Nanocellulose Composite Films for Giga/Terahertz Electromagnetic Shielding and Photothermal Conversion

**DOI:** 10.1007/s40820-024-01386-5

**Published:** 2024-04-08

**Authors:** Tian Mai, Lei Chen, Pei-Lin Wang, Qi Liu, Ming-Guo Ma

**Affiliations:** 1 Research Center of Biomass Clean Utilization, MOE Engineering Research Center of Forestry Biomass Materials and Bioenergy, Beijing Key Laboratory of Lignocellulosic Chemistry, College of Materials Science and Technology, Beijing Forestry University, Beijing, 100083 People’s Republic of China; 2 State Silica-Based Materials Laboratory of Anhui Province, Bengbu, 233000 People’s Republic of China

**Keywords:** Metal–organic frameworks, MXene, Nanocellulose, Electromagnetic shielding, Photothermal conversion

## Abstract

With the continuous advancement of communication technology, the escalating demand for electromagnetic shielding interference (EMI) materials with multifunctional and wideband EMI performance has become urgent. Controlling the electrical and magnetic components and designing the EMI material structure have attracted extensive interest, but remain a huge challenge. Herein, we reported the alternating electromagnetic structure composite films composed of hollow metal–organic frameworks/layered MXene/nanocellulose (HMN) by alternating vacuum-assisted filtration process. The HMN composite films exhibit excellent EMI shielding effectiveness performance in the GHz frequency (66.8 dB at Ka-band) and THz frequency (114.6 dB at 0.1–4.0 THz). Besides, the HMN composite films also exhibit a high reflection loss of 39.7 dB at 0.7 THz with an effective absorption bandwidth up to 2.1 THz. Moreover, HMN composite films show remarkable photothermal conversion performance, which can reach 104.6 °C under 2.0 Sun and 235.4 °C under 0.8 W cm^−2^, respectively. The unique micro- and macro-structural design structures will absorb more incident electromagnetic waves via interfacial polarization/multiple scattering and produce more heat energy via the local surface plasmon resonance effect. These features make the HMN composite film a promising candidate for advanced EMI devices for future 6G communication and the protection of electronic equipment in cold environments.

**Supplementary Information:**

The online version contains supplementary material available at 10.1007/s40820-024-01386-5.

## Highlights

- The composite films are composed of hollow metal–organic frameworks/layered MXene/nanocellulose with unique alternating electromagnetic structures.
- The optimized composite films exhibit excellent EMI shielding performance of 66.8 dB at GHz frequency and 114.6 dB at THz frequency.
- The EMI shielding ability of composite films to electromagnetic waves is verified by practical visualized application simulation.
- The composite films show remarkable photothermal conversion performance, which can reach 235.4 °C under 0.8 W cm^−2^.

**Supplementary Information:**

The online version contains supplementary material available at 10.1007/s40820-024-01386-5.

## Graphical abstract

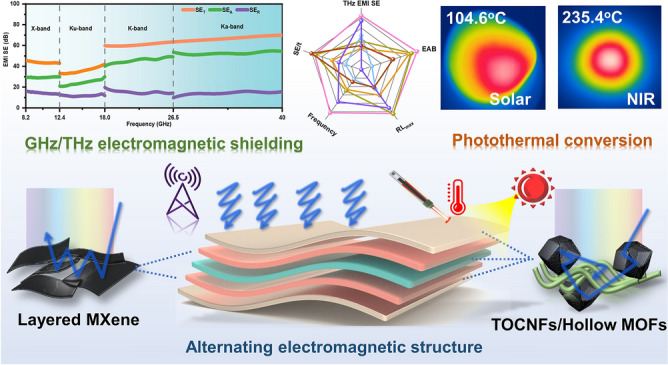

## Introduction

With the rapid development of modern science and technology, electronic devices have become ubiquitous in human daily life. However, electronic equipment brings convenience to our lives but also produces electromagnetic radiation pollution [1, 2]. As long as the electronic equipment is operating, it will produce electromagnetic radiation and affect the normal operation of the equipment, endangering human life and health [3, 4]. Especially in the 6G era, the frequency of electromagnetic wave bands used in communication expanded from GHz to THz, which undoubtedly aggravated the electromagnetic radiation pollution in the environment [5]. So, the development of electromagnetic protection materials is particularly important. Recently, cellulose-based electromagnetic protection materials have attracted wide attention [6]. Because of the abundant hydroxyl groups on the surface of cellulose, different functional groups can be obtained by modifying the hydroxyl group or cross-linking with functional materials through hydrogen bonding [7, 8]. Therefore, the selection of appropriate functional materials is the key to improving cellulose-based electromagnetic protection materials.

MXene was a new family in 2D material, which was first reported by Gogotsi's group in 2011 [9]. MXene has been found to have outstanding electromagnetic shielding performance due to its excellent electrical conductivity (≥ 10 times of reduced graphene oxide films) [10]. However, the development of MXene in the field of electromagnetic shielding materials is limited by a single electrical loss mechanism, which is limited by weak absorption attenuation, excessive conductivity, insufficient magnetic loss, and poor impedance matching performance [11]. Therefore, the development of electromagnetic shielding materials based on the electromagnetic double-loss mechanism is particularly important [12]. Metal–organic frameworks (MOFs) have the characteristics of low density, ordered structure, and rich adjustability. The metal nodes in MOFs will be reduced to metal nanoparticles, and the ligands will be converted into graphitized carbon after pyrolysis [13]. More importantly, due to the ordered structure, the metal nanoparticles will be doped in the graphitized carbon framework in a uniform distribution to avoid aggregation after pyrolysis. Therefore, MOFs-derived carbon materials are considered as excellent electromagnetic shielding materials. MOFs with high permeability can complement the advantages of MXene with high conductivity [14]. However, doping lower conductive MOFs fillers directly into high conductive MXene will block the original conductive path, resulting in a sharp decline in conductivity, thus limiting the EMI performance of electromagnetic shielding materials [15].

The structural design of composite materials is also considered another effective way to adjust the impedance matching of electromagnetic waves at the interface by multiple reflections and scattering, thus improving the performance of EMI shielding effectiveness (EMI SE) and absorption efficiency [16]. Cao et al*.* [17] developed the carbon nanotubes/MXene/2,2,6,6-tetramethylpiperidine-1-oxyl oxidated cellulose nanofiber (TOCNFs) composite paper via a facile alternating vacuum-assisted filtration process with the EMI SE of 34.8 dB in the X band. The gradient sandwich structure regulates the contributions from reflection and absorption to effectively improve the performance of electromagnetic wave absorption efficiency of materials. Hu et al. [18] reported the asymmetric gradient structure CNFs/reduced graphene oxide/Fe_3_O_4_/silver nanowires nanocomposite film with the EMI SE from 63.1 to 112.9 dB in the X band. The unique asymmetric gradient structure can endow the composite with higher EMI SE via gradient absorption and gradient reflection in the layers. These works have greatly promoted the development of electromagnetic shielding films in structural design. However, the GHz and THz EMI research of multifunctional MOFs/MXene/nanocellulose composite films with unique gradient alternating electromagnetic structures have rarely been reported.

In this work, using TOCNFs as the polymer matrix, we fabricated MOFs-derived cobalt hollow carbon cage (Co-HCC) via in situ epitaxial growth and pyrolysis. Then, the hollow MOFs/layered MXene/nanocellulose (HMN) composite films with alternating electromagnetic structures were designed by the alternating vacuum-assisted filtration (AVAF) method. We explored that the alternating electromagnetic structure endows the GHz and THz electromagnetic wave transmission with a unique "alternating absorption-reflection" process, effectively improving the electromagnetic shielding performance. In addition, the practical EMI applications simulation of the HMN composite films was also investigated. More importantly, the excellent photothermal conversion ability also endows HMN composite films with the reliability of keeping electronic devices operating in cold environments. This work proves that MOFs/MXene/nanocellulose composite films with alternating electromagnetic structures have potential applications in electronic warfare and electronic equipment anti-interference field.

## Experimental Section

### Materials

TEMPO-oxidized cellulose nanofibers (TOCNFs, 0.2 wt%) dispersion was purchased from Tianjin woodelfbio Co., Ltd. MAX Ti_3_AlC_2_ (99%, 400 mesh) was purchased from Jilin 11 Technology Co., Ltd. Lithium fluoride (LiF, 99%), 2-methylimidazole (C_4_H_6_N_2_, 98.0%), cetyltrimethylammonium bromide (CTAB, 99%), and *N*,*N*-dimethylformamide (DMF, AR) were purchased from Shanghai Macklin Biochemical Technology Co., Ltd. Cobaltous nitrate hexahydrate (AR) and zinc nitrate hexahydrate (AR) were obtained from Guangzhou Huada Chemical Reagent Co., Ltd. Methanol (AR) and concentrated hydrochloric acid (HCl) were supplied by Beijing Modern Oriental Fine Chemistry Co., Ltd. The above chemicals were used without further purification. Filter membranes (cellulose, 0.45 μm pore size) were supplied by Tianjin Jinteng Experiment Equipment Co., Ltd.

### Synthesis of Hollow Metal–Organic Frameworks

The synthesis process is based on a previous report [19]. For the synthesis of ZIF-8, the 0.02 mol zinc nitrate hexahydrate and 0.075 mol C_4_H_6_N_2_ were dissolved in 100 mL methanol, respectively. The C_4_H_6_N_2_ then slowly dripped into the zinc nitrate solution hexahydrate solution. The solution was mixed and stirred at room temperature for 24 h. Subsequently, the white precipitate was collected by centrifugation with methanol and DMF several times to remove the remaining chemicals and replace fresh solvents each time, followed by vacuum-drying at 80 °C overnight. For the synthesis of ZIF-67, 0.02 mol cobalt nitrate hexahydrate and 0.075 mol C_4_H_6_N_2_ were dissolved in 100 mL methanol, respectively. The subsequent processing method is similar as the synthesis of ZIF-8. For the synthesis of ZIF-8@ZIF-67, 0.5 g ZIF-8 was dispersed in 100 mL methanol solution containing 0.02 mol cobalt nitrate hexahydrate. Then, 100 mL methanol solution containing 0.075 mol C_4_H_6_N_2_ was further slowly dripped into the above mixture. The subsequent processing method is similar as the synthesis of ZIF-8. The pyrolysis process was carried out in a tube furnace at a heating rate of 2 min^−1^ to 800 °C (maintained at 800 °C for 2 h) under nitrogen atmosphere to obtain the Co–HCC nanoparticles.

### Synthesis of Delaminated Ti_3_C_2_T_*x*_ MXene Nanosheets

Specifically, 1 g of LiF was added in 20 mL diluted HCl (9 M). Then, 1 g of Ti_3_AlC_2_ was slowly added into the HCl solution and kept stirring at 36 °C for 48 h and centrifuged with deionized water several times to obtain the multiple layers of Ti_3_C_2_T_*x*_ (m-Ti_3_C_2_T_*x*_). Finally, the m-Ti_3_C_2_T_*x*_ was exfoliated via ultrasound under the ice bath and then centrifuged again. The obtained product is the delaminated Ti_3_C_2_T_*x*_ (d-Ti_3_C_2_T_*x*_).

### Preparation of Hollow Metal Organic Frameworks/MXene/Nanocellulose (HMN) Composite Films

HMN composite films were prepared by the alternating vacuum-assisted filtration (AVAF) method. First, the surface of Co-HCC was modified with cationic surfactant CTAB (10 wt% based on Co-HCC) by mechanical stirring for 30 min [20]. Then, the dried Co-HCC was dispersed in TOCNFs suspension with ultrasonicated and mechanical stirred well-distributed. Herein, the mass ratio of TOCNFs and Co-HCC is controlled at 2:1. Then, the homogeneously suspended solution was vacuum-filtrated onto a cellulose filter membrane to form stable films. Subsequently, the d-Ti_3_C_2_T_*x*_ suspension was vacuum filtrated on top of TOCNFs/Co-HCC to form stable films. Then repeat the above vacuum-filtrated steps to construct the gradient alternating electromagnetic structure. The HMN composite films with different total layers and d-Ti_3_C_2_T_*x*_ content are named HMN-mL-*n*% (m means the total number of layers, and *n* means the d-Ti_3_C_2_T_*x*_ content). The component content of each layer controls the gradient ratio change. For example, HMN-5L-57.1 wt% means that the total number of layers of composite films is five, including two d-Ti_3_C_2_T_*x*_ layers and three TOCNFs/Co-HCC layers. The composition table is displayed in Tables S4–S7. After vacuum filtration, the obtained HMN composite films were dried at 50 °C in vacuum oven overnight and then carefully peeled off.

### Characterization

The microstructure of composites was investigated by scanning electron microscope (SEM, ZEISS Gemini SEM 300, Germany), transmission electron microscope (TEM, JEM-2100 Plus, Japan) equipped with an energy-dispersive spectroscopy (EDS). The infrared spectra of samples were measured on a Fourier transform infrared spectrometer (FTIR, Thermo Scientific Nicolet iS5, USA). The surface chemistries of the samples were characterized by X-ray photoelectron spectroscopy (XPS, Thermo Scientific K-Alpha, USA). Raman spectra were performed on a laser Raman spectrometer with a 633-nm line laser as the excitation source (Horiba LabRAM HR Evolution, Japan). The phase structures of the composites were characterized by X-ray diffractometer (XRD, Rigaku Ultima IV, Japan). Thermal gravimetric analysis (TGA) was performed using a thermogravimetric analyzer (TA STD Q600, USA). Brunauer–Emmett–Teller (BET, ASAP 2460, USA) analysis measured the specific surface area. Zeta-potentials (Malvern Zetasizer Nano ZS90, Britain) were measured in de-ionized water. The electromagnetic radiation detector (LZT-1000, China) was used to detect the electromagnetic radiation of the Tesla coil. The electrical conductivity was tested with Suzhou Jingge Electronic Four Probe Tester (ST2258C, China). The mechanical performance of materials was tested by an electronic universal testing machine (Instron 5943, USA). The tensile samples (20 mm long and 10 mm wide) were tested at a constant tensile rate of 0.2 mm min^−1^. The magnetic hysteresis loop was measured by the vibrating sample magnetometer (VSM, BKT-4700, China). The relative permeability was calculated as follows:

1 $$\mu_{r} = \frac{(\& + 1)}{{\mu_{0} }}$$

where &, *μ*_*r*_ and *μ*_*0*_ are magnetic susceptibility, relative permeability, and vacuum permeability. *μ*_0_ = 4π × 10^−7^ H m^−1^.

The EMI shielding effectiveness (EMI SE) of samples was measured on the vector network analyzer (Agilent PNA-N5244A, USA) based on the S parameters. All composite films were cut to the size suitable for the test mold. For the frequency of 8.2–12.4 GHz, the sample size is 22.9 × 10.20 mm^2^. For the frequency of 12.4–18 GHz, the sample size is 15.9 × 8.03 mm^2^. For the frequency of 18.0–26.5 GHz, the sample size is 10.95 × 4.50 mm^2^. For the frequency of 26.5–40 GHz, the sample size is 7.15 × 3.60 mm^2^. Then the cut sample is firmly fixed in the cavity of the test mold. All the powder samples were mixed with melted paraffin, and the samples with suitable dimensions (inner 3.04 mm, outer 7.0 mm) were prepared at the mass ratio of powder to paraffin of 1:3 and tested in 8.2–12.4 GHz. Then the mixed paraffin sample is firmly fixed in the cavity of the test mold. The power of the vector network analyzer is set as − 3 dBm, and the intermediate frequency bandwidth is set as 300 Hz.

The GHz EMI SE was calculated as follows:

2 $${\text{SE}}_{{\text{T}}} = {\text{SE}}_{{\text{R}}} {\text{ + SE}}_{{\text{A}}}$$

3 $${\text{SE}}_{{\text{T}}} = {10 } \times {\text{log}}_{{{10}}} \left( {\frac{{1}}{{\left| {S_{{{21}}} } \right|^{{2}} }}} \right)$$

4 $${\text{SE}}_{{\text{R}}} = {10 } \times {\text{ log}}_{{{10}}} \left( {\frac{{1}}{{{1 } - \left| {S_{{{11}}} } \right|^{{2}} }}} \right)$$

5 $${\text{SE}}_{{\text{A}}} = {10 } \times {\text{ log}}_{10} \left( {\frac{{{1 } - \left| {S_{{{11}}} } \right|^{{2}} }}{{\left| {S_{{{21}}} } \right|^{{2}} }}} \right)$$

6 $${\text{SE/}}t = \frac{{\text{EMI SE}}}{{{\text{thickness}}}} ={\text{ dB}} \upmu {\text{m}}^{{{\text{{-}1}}}}$$

7 $${\text{SE/}}t = \frac{{\text{EMI SE}}}{{{\text{thickness}} \times {\text{density}}}} = {\text{dB cm}}^{{2}} {\text{ g}}^{{{\text{{-}1}}}}$$

where SE_T_, SE_R_, and SE_A_ are the GHz EMI SE, absorption of EMI SE, and reflection of EMI SE, respectively. SE/*t* is the thickness specific shielding effectiveness. SSE/*t* is specific shielding effectiveness [21, 22].

Terahertz time-domain spectroscopy measured the THz signals in the transmission mode system (Teraview TeraPulse Lx, Britain) and reflection mode system (Advantest TAS7400TS, Japan). The terahertz imaging was obtained by TAS7 × 00 2D Mapping Excel Macro R1.03. All composite films were cut to the size of 20 × 20 mm^2^ suitable for the test mold and placed on a THz hollow circular (pore diameter is 12 mm) sample placement. The THz EMI SE was calculated as follows:

8 $${\text{THz EMI SE}} = - {20} \times {\text{log}}_{{{10}}} \left( {\frac{{E_{{\text{s}}} }}{{E_{{\text{a}}} }}} \right)$$

9 $$R_{{\text{L}}} = - 20 \times \log_{10} \left( {\frac{{E_{{\text{r}}} }}{{E_{i} }}} \right)$$

where *E*_s_, *E*_a_, *R*_L_, *E*_r_, and *E*_i_ are transmission terahertz electric field intensity for the samples, transmission terahertz electric field intensity for the air cavity, terahertz reflection loss, the amplitude intensity of the reflection signal of the samples, the amplitude intensity of the reflection signal of the aluminum mirror [23].

The UV–Vis–NIR absorbance spectrum was obtained by the UV–visible-near-infrared spectrophotometer (Shimadzu UV-3600i Plus, Japan). The infrared emissivity was tested by infrared emissivity tester (TSS-5X, Japan). The surface temperature of the samples was monitored by the infrared thermal imaging camera (Fotric 226, China). The 808-nm NIR laser (Shanghai Connect Fibre Optics Co., Ltd, China) and a Xenon lamp (PL-X500D, Beijing Precise Technology Co., Ltd, China) were used in experiment. The solar-to-thermal energy conversion efficiencies (*η*) of the samples were calculated as follows:

10 $$\eta = \frac{{H_{{{\text{in}}}} - H_{{{\text{loss}}}} }}{{H_{{{\text{in}}}} }} = \alpha - \frac{{\varepsilon \sigma (T^{4} - T_{{\text{r}}}^{4} )}}{{I_{{{\text{solar}}}} }}$$

where *H*_in_ is the energy input, *H*_loss_ is the energy loss, the *α* is the solar absorptivity, the *ε* represents the infrared emissivity, the *T* is the temperature of HMN composite films and *T*_r_ is room temperature, the *σ* points the Stefan–Boltzmann constant, the *I*_solar_ represents the power density of the incident sunlight [24].

## Results and Discussion

### Synthesis and Microstructure of HMN Composite Films

**Fig. 1:**
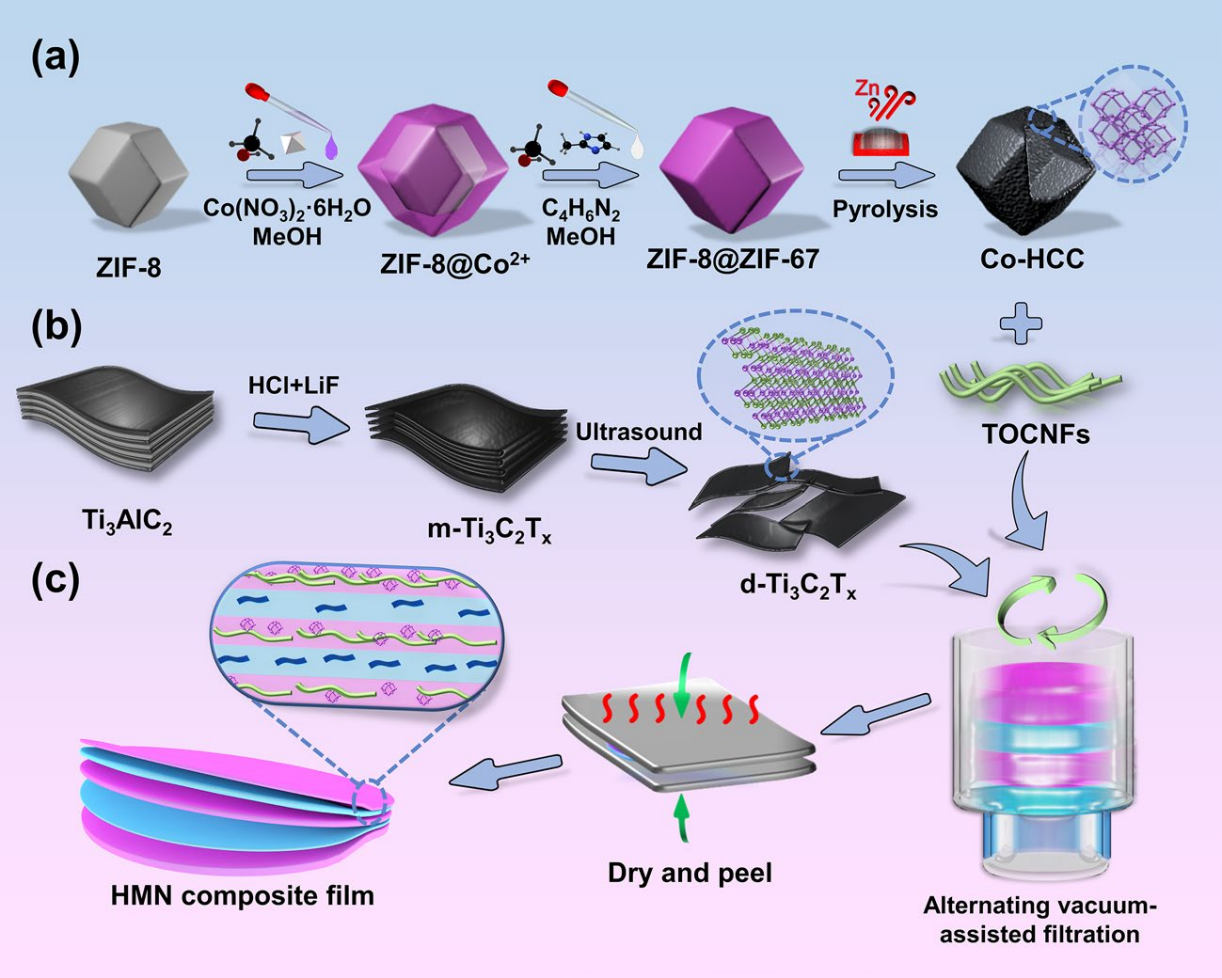
Schematic diagrams of synthesis of HMN composite films by AVAF strategy. The fabrication process of **a** Co-HCC, **b** d-Ti_3_C_2_T_*x*_, and **c** HMN composite films

The synthesis of HMN composite films is illustrated in Fig. 1. Generally speaking, the hollow Co-HCC was mixed with TOCNFs suspension and then vacuum-filtrated on the cellulose filter membrane. After that, the d-Ti_3_C_2_T_*x*_ suspension was vacuum-filtrated on top of TOCNFs/Co-HCC. Finally, the above step is repeated repeatedly until stable HMN composite films are formed. Specifically, the ZIF-8@ZIF-67 with shell-core structure was prepared by *in situ* epitaxial growth. The ZIF-8 was used as the core, attracting Co^2+^ on its surface through electrostatic interaction (Fig. 1a). Organic ligand C_4_H_6_N_2_ was then added to facilitate the growth of ZIF-67 on the surface. As shown in Fig. S1, ZIF-8@ZIF-67 displays the classic rhombic dodecahedron structure as the same as ZIF-8. The particle size of ZIF-8@ZIF-67 (about 1.4 μm) is larger than that of ZIF-8 (about 1.0 μm), attributed to the epitaxial growth of ZIF-67 on the surface of ZIF-8. For further evidence of the shell-core structure of the as-prepared ZIF-8@ZIF-67, EDS line scanning reveals that Zn resides predominantly in the “central core,” while Co is mainly concentrated in the “outer shell” (Fig. S2). The unique core–shell structure makes ZIF-8@ZIF-67 form a hollow structure after pyrolysis. To avoid excessive collapse of the ZIF-8@ZIF-67 structure, 800 °C was selected as the calcination temperature [25, 26]. The imidazole ligand of ZIF-8@ZIF-67 was transformed into highly graphitized carbon, and the cobalt metal nodes of ZIF-67 were reduced. Due to the low boiling point of zinc, metal nodes within ZIF-8 will be gradually selectively evaporated away at high temperature (~ 800 °C) from the “central core” during pyrolysis [27].

**Fig. 2:**
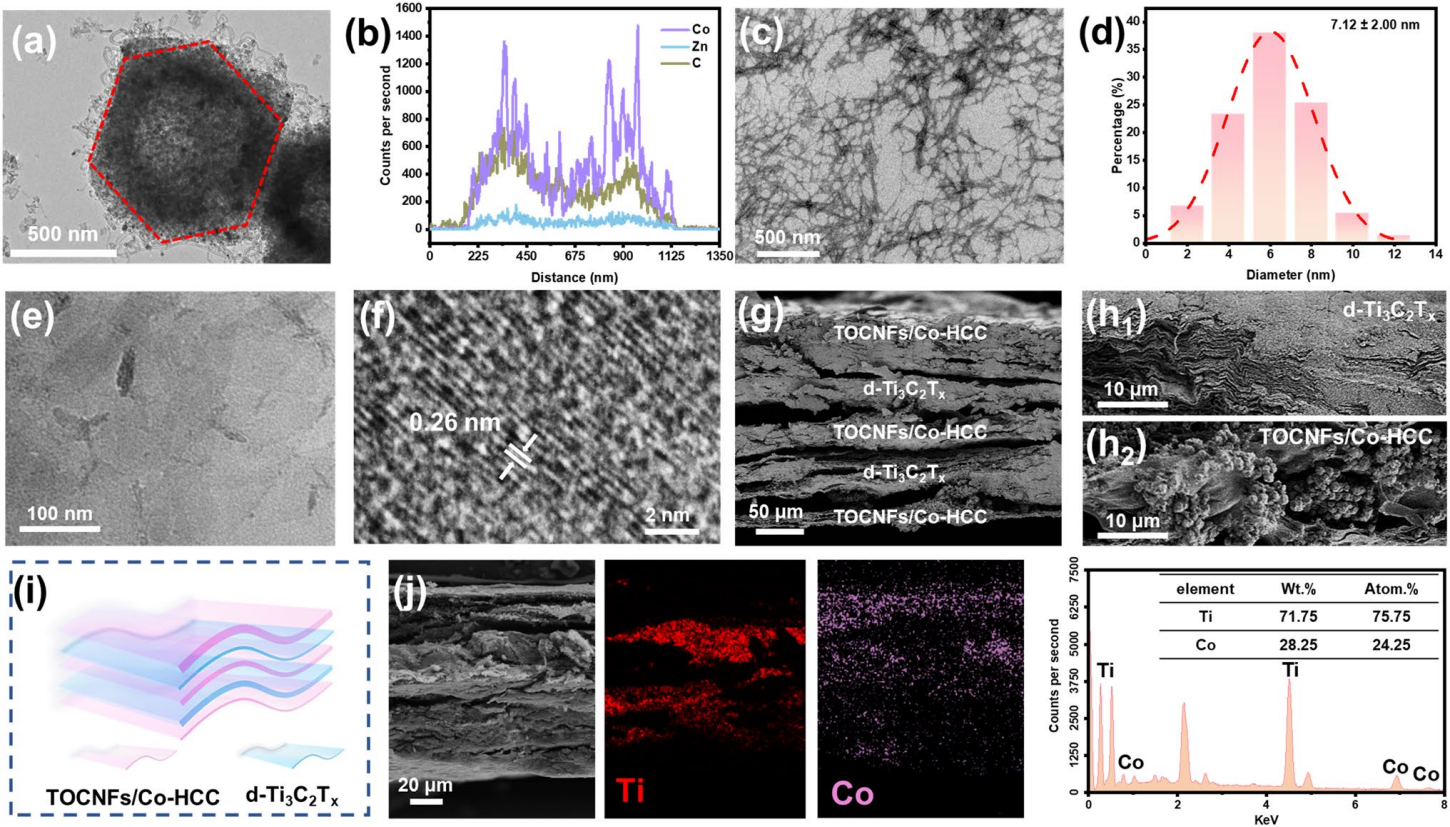
Microstructure of the HMN composite films. **a** TEM image and **b** EDS line scanning image of Co-HCC, respectively. **c** TEM image and **d** size distribution of TOCNFs, respectively. **e** TEM image and **f** lattice fringe of d-Ti_3_C_2_T_*x*_ nanosheets, respectively. **g, h** SEM image, **i** schematic illustration, and **j** EDS mapping of HMN composite films

As shown in Fig. 2a, the Co-HCC shows the shape of the hollow polyhedron with the size of about 1.2 μm, and the shrinkage of the size is caused by the inevitable partial collapse of the ZIFs material during pyrolysis, which is consistent with the results reported in the previous literature [28]. During the pyrolysis at high temperature, the flowing gas promotes the outward movement of metal ions and the formation of the hollow polyhedron. Besides, many CNTs are anchored on the rough surface of Co-HCC. The formation of CNTs on the surface is mainly due to the catalysis of Co nanoparticles and the evaporation of Zn [29]. Correspondingly the 0.20 nm lattice fringe with a spacing was assigned to the (111) crystal face of Co, and 0.36 nm was assigned to the (002) crystal face of highly graphitized carbon (Fig. S3) [30]. Furthermore, as displayed in Figs. 2b and S4, the EDS line scanning image and mapping exhibited the hollow structure of Co-HCC, Co is mainly distributed in the “outer shell,” and the “hollow core” is the graphite carbon of the organic ligand from carbonized ZIF-8. In addition, the signal intensity of the Zn element is slight in the whole image, which further proves that Zn is evaporated during pyrolysis. Moreover, Co-HCC also shows obvious magnetism and can be attracted by magnets (Fig. S5a). TEM images show that the diameter of the TOCNF measures approximately 7.12 ± 2.00 nm, whereas its length is to be at the micrometer level (Fig. 2c, d). The high aspect ratio can easily lead to physical entanglement between TOCNFs and form a stable structure [31]. The diagram of the fabrication process of d-Ti_3_C_2_T_*x*_ is displayed in Fig. 1b. A mild etching method was employed using LiF and hydrochloric acid as etchants to selectively etch the Al layer in the MAX Ti_3_AlC_2_ phase, resulting in the formation of accordion-shaped m-Ti_3_C_2_T_x_, which was then further processed to obtain d-Ti_3_C_2_T_*x*_ through ultrasonic delaminated (Fig. 2e, f). Moreover, both the suspension of d-Ti_3_C_2_T_*x*_ and TOCNFs displayed a distinct Tyndall effect when exposed to laser pointer irradiation, indicating the colloidal nature of the solutions prepared (Fig. S5b, c). In the AVAF process, Co-HCC was modified with cetyltrimethylammonium bromide (CTAB) and then dispersed into a TOCNFs suspension. After CTAB modification, the electro-positivity of Co-HCC is improved, which is helpful in generating electrostatic interaction with TOCNFs and d-Ti_3_C_2_T_*x*_ to connect them closely (Fig. S6) [32]. Next, the resulting TOCNFs/Co-HCC solution was stirred and ultrasound to full uniformity. The TOCNFs/Co-HCC mixture was then vacuum-filtered onto a cellulose filter membrane. The d-Ti_3_C_2_T_*x*_ solution is then vacuum-filtered at the top of TOCNFs/Co-HCC. The above steps are repeated to form a stable film structure (Fig. 1c). Finally, the HMN composite films were dried and peeled off from the cellulose filter membrane. As seen from Fig. 2g–i, HMN composite films have apparent gradient alternating structure. The d-Ti_3_C_2_T_*x*_ presents the layered structure, while TOCNFs/Co-HCC presents the shish kebab structures. Besides, it can be seen from the EDS spectrum that the Ti element in d-Ti_3_C_2_T_*x*_ and the Co element in Co-HCC are mainly divided into each layer. There is slight infiltration between layers, which is formed by the close connection between different layers, which is helpful in forming a stable film structure (Fig. 2j). The results further prove that the AVAF method can form gradient alternating structures composite films, which helps to give play to the respective advantages of different components in a whole material.

### Characterization of Crystallographic Structure and Composition of the HMN Composite Films

**Fig. 3:**
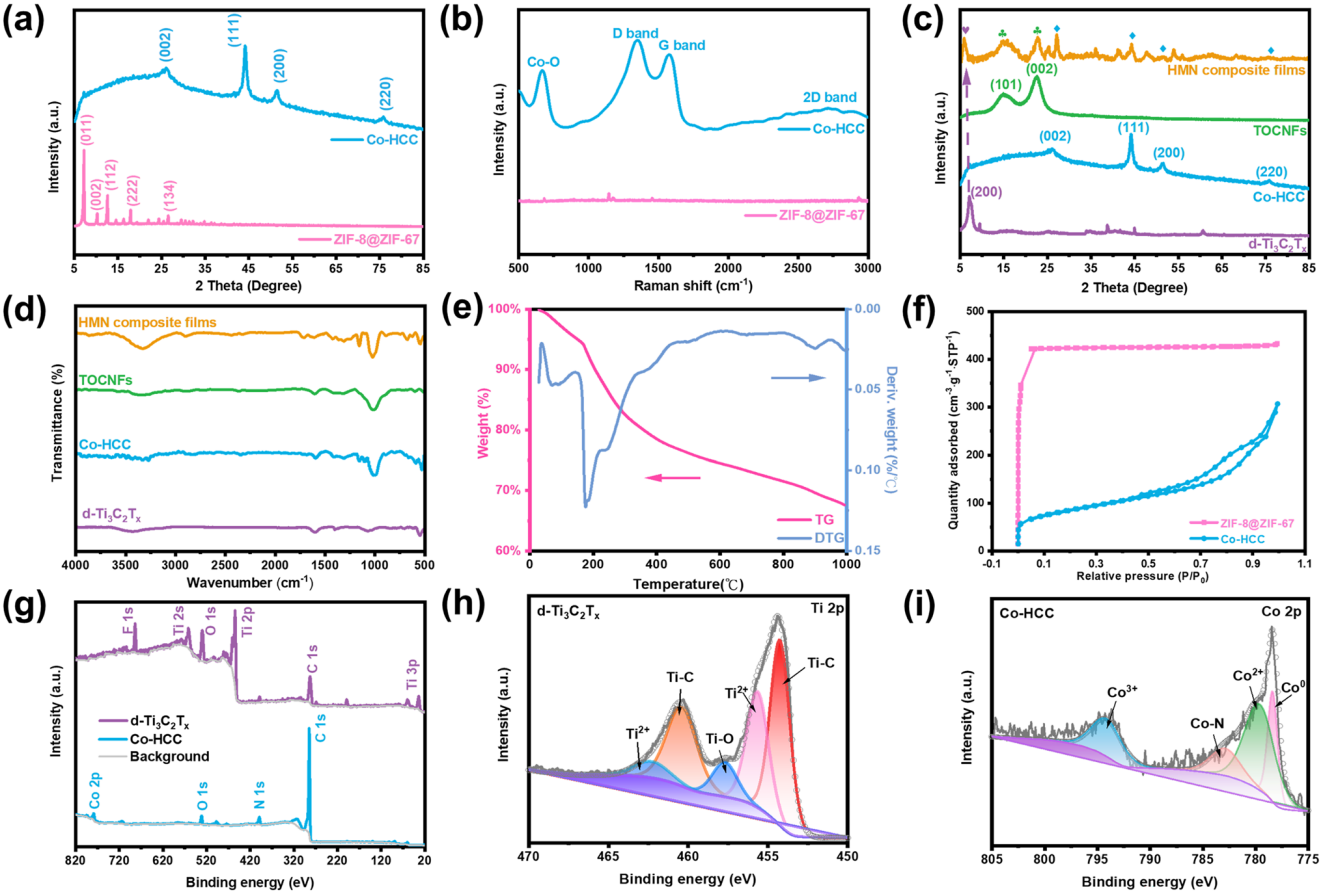
The crystallographic structure and composition of the HMN composite films. **a** XRD patterns and **b** Raman patterns of ZIF-8@ZIF-67 before and after pyrolysis, respectively. **c** XRD patterns and **d** FTIR spectra of HMN composite films, TOCNFs, Co-HCC, and d-Ti_3_C_2_T_*x*_, respectively. **e** TG and DTG analysis of HMN composite films. **f** The nitrogen adsorption–desorption isotherms of ZIF-8@ZIF-67 and Co-HCC. **g** XPS full spectra of d-Ti_3_C_2_T_x_ and Co-HCC. XPS fine spectra of **h** Ti 2*p* of d-Ti_3_C_2_T_*x*_ and **i** Co 2*p* of Co-HCC

The crystallographic structure and phase composition of the resultant HMN composite films were further explored. As shown in Fig. S7a, all ZIF-8@ZIF-67, ZIF-8, and ZIF-67 exhibit a series of similar characteristic peaks in the range of 5°–35°, which explains that ZIF-8@ZIF-67 with shell-core structure prepared by epitaxial growth method can effectively retain the crystal properties of ZIF-8 and ZIF-67 [33]. After pyrolysis, the XRD characteristic peaks of ZIF-8@ZIF-67 disappeared and transformed into three characteristic peaks (44.2°, 51.5°, and 75.9°) of Co corresponded to (111), (200), and (220) planes with cubic structure. Besides, the characteristic peak (26.6°) of graphite carbon has corresponded to (002) plane (Fig. 3a). These prove that the Co metal node in the shell ZIF-67 has been successfully reduced to metal Co, and the organic ligand of ZIF-8@ZIF-67 is transformed into graphite carbon, which is consistent with the previous report [34]. Additionally, as displayed in Fig. 3b, the phenomenon can also be proved by the new peaks (Co–O, D band, G band, and 2D band) of Co-HCC in the Raman spectrum, compared with ZIF-8@ZIF-67 [35]. As shown in Figs. 3c and S7b, from the XRD pattern, two characteristic diffraction peaks (2*θ* = 15.0 and 22.5°) were assigned to (101) and (002) crystal planes of TOCNFs. In addition, the characteristic (002) peak of d-Ti_3_C_2_T_*x*_ shifts from 2*θ* = 7.1° to 6.0°, proving the interconnection between TOCNFs/Co-HCC and d-Ti_3_C_2_T_*x*_ [36].

Besides, the above characteristic peaks are apparent in the HMN composite films, which prove the successful preparation of the composite materials. To further explore the components of HMN composite films, the FTIR spectrum is displayed in Fig. 3d. The characteristic peaks of TOCNFs were observed at 1050 and 1600 cm^−1^, attributing to the stretching vibration of the C–O–C group of the glucopyranose ring and the existence of the carbonyl [37]. The Ti_3_AlC_2_ where no particular peak can be detected in the FTIR spectrum, depicts the purity of MAX. The d-Ti_3_C_2_T_*x*_ shows peaks at 580 and 1380 cm^−1^ that correspond to –OH and C–F groups. Besides, all ZIF-67, ZIF-8, and ZIF-8@ZIF-67 present several characteristic peaks in the range of 500–2000 cm^−1^ (Fig. S8). After carbonization at high temperature, the two new peaks remain at 560 and 650 cm^−1^, corresponding to Co [38]. These characteristic peaks were also observed in the FTIR spectrum of HMN composite films. TGA and DTG were used to analyze the composition of HMN composite films (Fig. 3e). The decline curve of TG accords with cellulose. The high residual weight percentage is attributed to d-Ti_3_C_2_T_*x*_ derivative titanium dioxide at high temperature and Co-HCC [39, 40]. The specific surface areas and N_2_ absorption–desorption of ZIF-8@ZIF-67 and Co-HCC were researched (Figs. 3f and S9a). N_2_ absorption–desorption results show that saturation is achieved at low relative pressure for the ZIF-8@ZIF-67, demonstrating the microporous structure of the materials with the typical type I adsorption–desorption isotherms. The specific surface area of ZIF-8@ZIF-67 reached 1565.6 and 1862.9 m^2^ g^−1^ in the BET model and Langmuir model, respectively. By contrast, the isotherms of Co-HCC show a relatively low N_2_ absorption–desorption capacity and specific surface area (297.7 and 920.7 m^2^ g^−1^ in the BET model and Langmuir model, respectively), probably caused by partial collapse of the frame. The typical type IV and the H3 hysteresis loop of Co-HCC exhibited the generation of hollow structure of materials [41]. Moreover, the average pore size (1.71 nm) of ZIF-8@ZIF-67 was less than the average pore size (6.38 nm) of Co-HCC. The pore size and pore volume of Co-HCC increased and gradually deviated from the microporous structure, which proved the hollow structure of Co-HCC (Figs. S9b and S10). The XPS analysis was used to further investigate the chemical compositions of d-Ti_3_C_2_T_*x*_ and Co-HCC (Fig. 3g–i). The XPS spectra show that the d-Ti_3_C_2_T_*x*_ and Co-HCC contain C, O, Ti, F, and C, O, N, Co elements, respectively. In the Ti 2*p* spectrum, a series of characteristic peaks for Ti–C, Ti–O, and Ti^2+^ were detected, thereby corroborating the existence of surface partial oxidation on the d-Ti_3_C_2_T_*x*_ [42]. In the Co 2*p* spectrum, a series of characteristic peaks for Co^0^, Co^2+^, and Co^3+^ suggest the presence of metallic Co^0^, cobalt oxide, and cobalt tetroxide in the Co-HCC [40]. Besides, as shown in Fig. S11, the XPS fine spectrum of the N 1*s* peak was deconvolved into four peaks, including oxidized-N, graphitic N, Co–N, and pyridinic N, respectively. In addition, the rich N element is helpful in improving the electromagnetic shielding performance of the materials [43]. The XPS fine spectra of Zn 2*p* indicated a doublet peak fitted at 1022.0 and 1045.1 eV attributed to Zn 2*p*_3/2_ and Zn 2*p*_1/2_. The binding energy difference between these observed Zn 2*p*_3/2_ and Zn 2*p*_1/2_ peaks about 23.1 eV, which is the same as the standard reference value of the ZnO (Fig. S12) [44].

### Electromagnetic Properties and Giga/Terahertz Electromagnetic Shielding Performances of HMN Composite Films

**Fig. 4:**
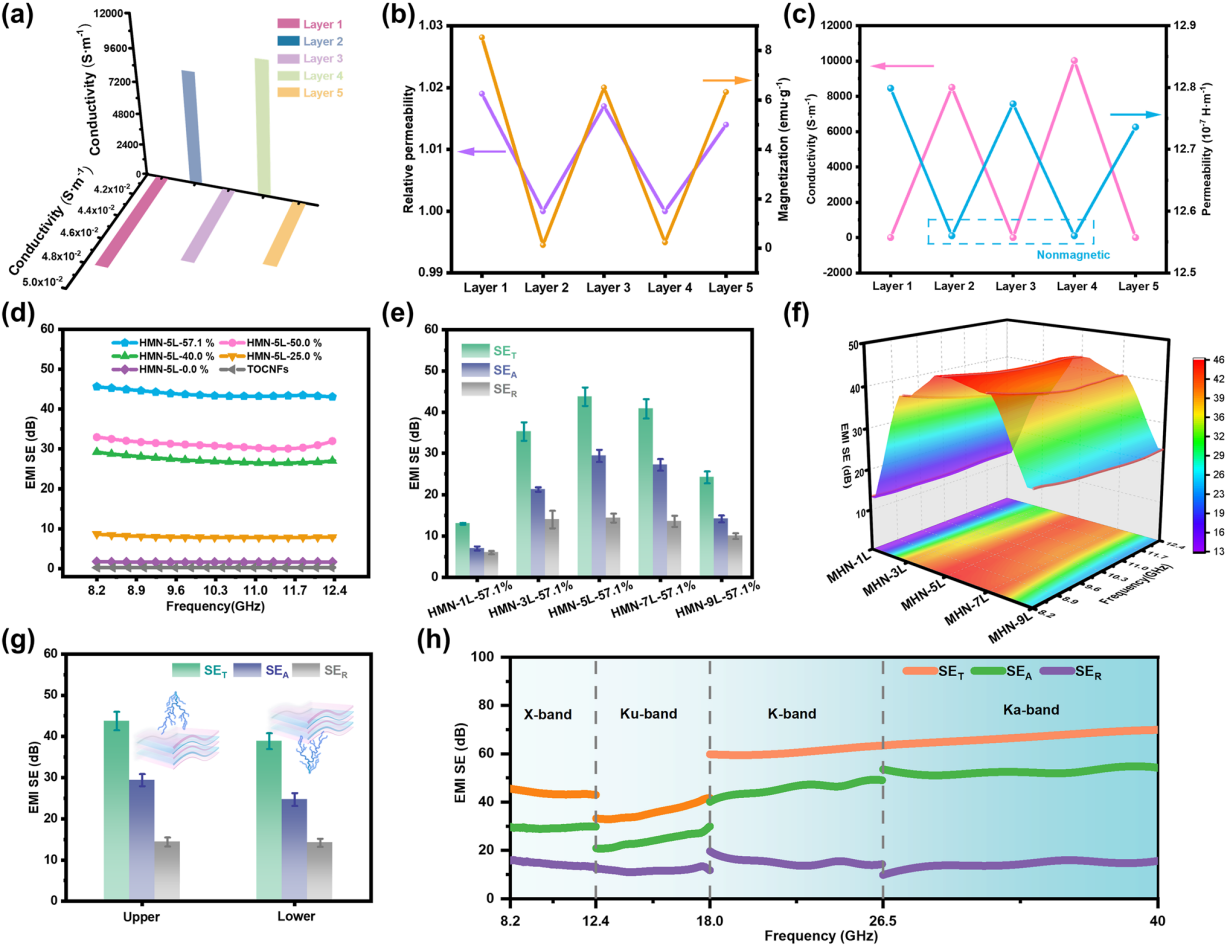
Electromagnetic properties and gigahertz electromagnetic shielding performances of HMN composite films. **a–c** Conductivity and magnetism of HMN-5L-57.1% composite films. **d** EMI SE of HMN composite films with different d-Ti_3_C_2_T_*x*_ contents. **e, f** EMI SE of HMN composite films with different numbers of layers. **g** Schematic diagram and EMI SE of HMN-5L-57.1% composite films when the electromagnetic waves are incident from different directions, respectively. **h** X, Ku, K, and Ka-band EMI SE of HMN-5L-57.1% composite films

Electrical conductivity and permeability are crucial in determining the EMI shielding properties. The electrical and magnetic characteristics of HMN composite films are shown in Figs. 4a, b and S13. Interestingly, the unique structural design endows the HMN composite films with unique electromagnetic structure because the contents of Co-HCC, MXene, and TOCNFs in each layer are different in the process of preparing HMN composite films and the electromagnetic properties of each layer will be different after TVAF process. Therefore, HMN composite films present an electromagnetic structure with gradient alternation. The total saturation magnetization of HMN composite film is 5.00 emu g^−1^. The first, third, and fifth layers are TOCNFs/Co-HCC layers with decreasing saturation magnetic induction (8.53, 6.50, and 6.32 emu g^−1^). The second and fourth layers are d-Ti_3_C_2_T_*x*_ layers with increasing electrical conductivity (8503.5 and 10,021.1 S m^−1^), which is greater than the minimum requirement for electromagnetic shielding performance (1 S m^−1^) [45]. As can be seen from Fig. 4c, the electrical conductivity and saturation magnetic induction of different layers in the composite film show an alternating increasing and decreasing trend. The conductive substance in the composite is mainly d-Ti_3_C_2_T_*x*_, while the magnetic substance is mainly Co-HCC nanoparticles. The saturation magnetization between different layers of HMN composite films shows an alternating trend of "strong–weak-strong–weak-strong," while the electrical conductivity shows an alternating trend of "low–high-low–high-low." This alternating electromagnetic structure contributes to the loss of electromagnetic waves inside the HMN composite films. The electromagnetic waves will produce interface loss between components with different conductivity and permeability, and orderly electromagnetic structure design components will also make it difficult for electromagnetic waves to penetrate the whole materials [46]. The vector network analyzer is used to measure the S parameters of GHz electromagnetic waves before and after passing through the samples (Fig. S14). To further prove the loss effect of materials on electromagnetic waves, the EMI SE of ZIF-8@ZIF-67, Co-HCC, and d-Ti_3_C_2_T_*x*_ was measured. ZIF-8@ZIF-67 exhibits negligible loss in its ability to mitigate electromagnetic waves due to the electrical and magnetic insulation properties. Conversely, Co-HCC exhibits a certain level of magnetic and electrical conductivity, thereby endowing it with the capacity to reflect and absorb electromagnetic waves. However, the relative lower conductivity impairs the EMI SE. The d-Ti_3_C_2_T_*x*_ displays exceptional conductivity, enabling it to reflect a significant proportion of electromagnetic waves. Consequently, d-Ti_3_C_2_T_*x*_ exhibits superior shielding performance while exhibiting a relatively low absorption value (Fig. S15). Therefore, structural design to fabricate composite materials comprising high-magnetic Co-HCC and high-conductive d-Ti_3_C_2_T_*x*_ is an approach that effectively enhances the overall performance of electromagnetic shielding materials. As can be seen in Fig. 4d, due to the high conductivity of d-Ti_3_C_2_T_*x*_, the electromagnetic shielding performance of HMN composite films is also improved with the increasing content of d-Ti_3_C_2_T_*x*_. When the content of d-Ti_3_C_2_T_*x*_ is increased from 0 to 57.1%, the EMI SE is also increased from 1.71 to 45.6 dB. However, pure TOCNFs films have almost no electromagnetic shielding performance (0.2 dB). Although the HMN-5L-0% composite films contained Co-HCC, the electromagnetic shielding performance is also low (1.71 dB) because of its poor conductivity. Therefore, conductivity plays a leading role in EMI SE in electromagnetic shielding materials, which is consistent with previous report [47]. To prove the influence of electromagnetic separation multi-layer structure on electromagnetic shielding performance, HMN-nL-57.1% with a total number of *n* (*n* = 1, 3, 5, 7, 9) layers was designed according to gradient content (Fig. 4e, f). When all the components are mixed to form a random structure, the average EMI SE of HMN-1L-57.1% is only 13.0 dB, which cannot meet the lowest standard of commercial electromagnetic shielding materials (more than 20 dB) [48]. With the increase in the number of layers, the average EMI SE of HMN-3L-57.1% and HMN-5L-57.1% can reach 35.3 and 43.7 dB, respectively.

After the structural design, the electromagnetic shielding performance of HMN-5L-57.1% is 3.36 times that of HMN-1L-57.1% without changing the total composition of the composite films. Interestingly, with the further increase of the number of layers, the average EMI SE of HMN-7L-57.1% and HMN-9L-57.1% is 40.8 and 24.2 dB, respectively. The reason for this situation may be that when there are too many layers, the thickness between each layer is too low, which makes the mutual penetration of the electromagnetic components more serious, leading to the decline of electromagnetic shielding ability [49]. However, it is still greater than the electromagnetic shielding ability of HMN-1L-57.1% with random structure. In addition, the design of the gradient structure also endows HMN composite films with different electromagnetic shielding abilities on two sides. As shown in Fig. 4g, the electromagnetic shielding ability of forward incidence is higher than that of backward incidence, which is attributed to the result of different reflection and absorption of electromagnetic waves on the surfaces of different layers. This phenomenon can be attributed to that when electromagnetic waves are incident from different layers of composite materials, it will affect the loss of electromagnetic waves in subsequent components, thus making the electromagnetic shielding performance different. Similar results have appeared in previous reports [50, 51]. Besides, to demonstrate the superiority of structural design, the electromagnetic shielding performance of MN composite films (without Co-HCC) and pure MXene films was evaluated (Figs. S16 and S17). The EMI SE and A coefficient of the HMN composite films were higher than MN composite films. This phenomenon further proves that the synergistic effect of Co-HCC magnetic loss and TOCNF scattering loss in the HMN composite films can improve the electromagnetic shielding performance [38]. The EMI SE of pure MXene films was slightly higher than HMN composite films, but the A coefficient was lower than HMN composite films. This is because pure MXene films have higher conductivity, which makes it have higher electromagnetic shielding ability, but it also brings the problem of impedance mismatch [31]. In order to further study the broadband EMI shielding performance of HMN composite films, the test electromagnetic frequency is extended to 40.0 GHz, including X-band (8.2–12.4 GHz), Ku-band (12.4–18 GHz), K-band (18–26.5 GHz), and Ka-band (26.5–40 GHz). As shown in Fig. 4h, HMN-5L-57.1% shows excellent average EMI SE in X-band (43.7 dB), Ku-band (36.0 dB), K-band (60.9 dB), and Ka-band (66.8 dB), which is beneficial to the individual requirements of electromagnetic shielding bands in different fields. Furthermore, the HMN composite films exhibited high absolute shielding effectiveness and thin thickness reaches up to 6534.1 dB cm^2^ g^−1^ and ~ 0.2 mm, which is envisaged for application within the electromagnetic shielding field of compact and mobile electronics apparatus (Fig. S18). In addition, the long-term stability of HMN composite films was also measured. Even after being stored at room temperature for 240 h, HMN composite films can still maintain 84.3% electromagnetic shielding performance (Fig. S19).

**Fig. 5:**
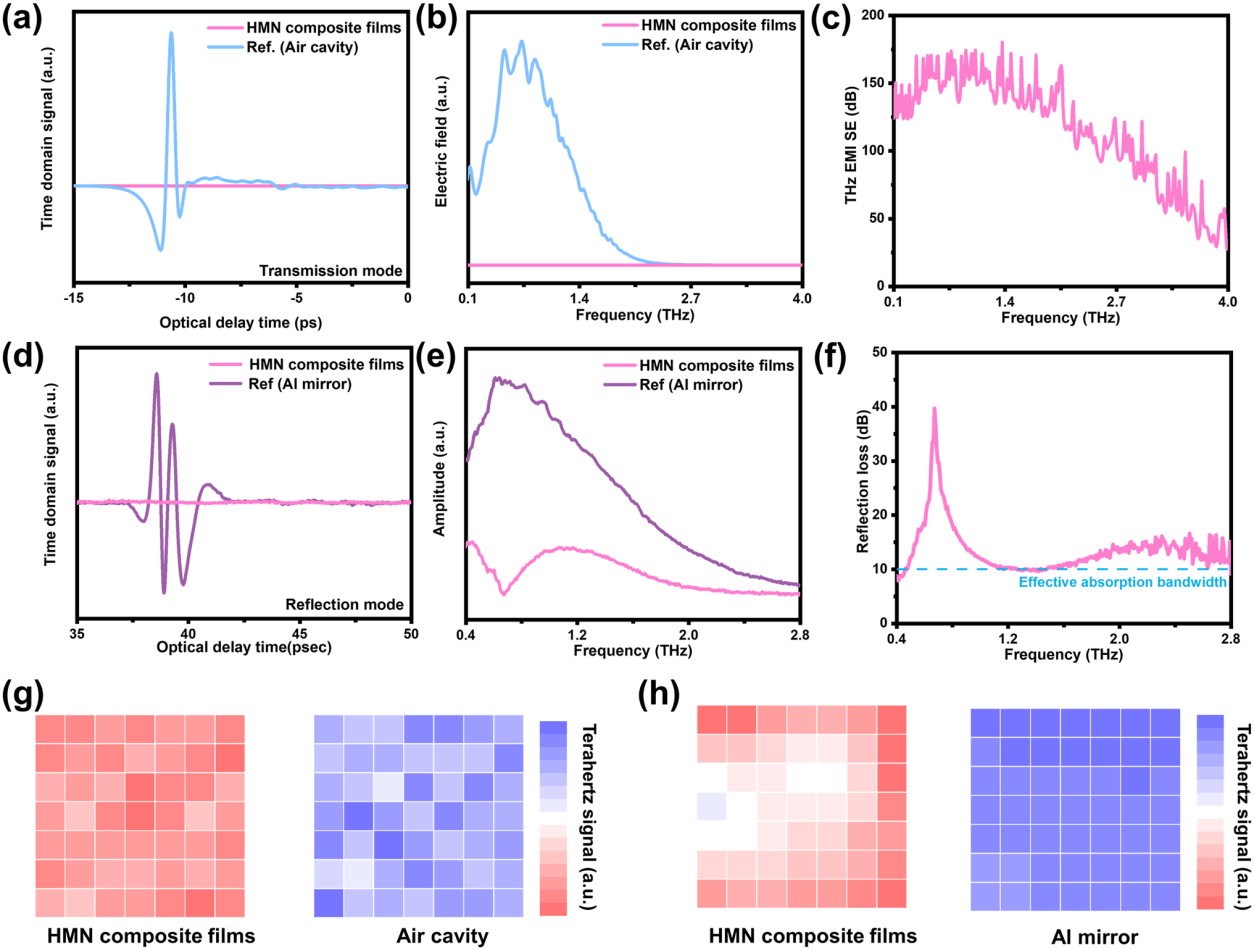
Terahertz electromagnetic shielding performances of HMN composite films. **a, d** THz time domain pulses for air, Al mirror, and HMN composite films in transmission model and reflection model. **b, e** Electric field intensity for air, Al plate, and HMN composite films in transmission model and reflection model. **c, f** EMI SE and reflection loss of HMN composite films in transmission model and reflection model. **g, h** Terahertz imaging thermogram of transmission mode and reflection mode

THz as an alternative electromagnetic wave band for future 6G communication, has higher frequency and energy, and its radiation to the human body cannot be ignored [52]. The electromagnetic shielding ability of HMN composite film at THz frequency is also researched. Terahertz time-domain spectroscopy is used to measure the signal and pattern changes of THz electromagnetic waves before and after passing through/reflecting the samples (Fig. S20). As displayed in Fig. 5a, b in transmission mode, terahertz waves undergo significant attenuation when passing through HMN composite films, which proves that HMN composite films have strong shielding performance for terahertz waves. Because of the signal-to-noise ratio, porosity of the material, and the special functional relationship between the SE and frequency, the EMI SE part fluctuates in the whole THz range [18, 53, 54]. However, within the range of 0.1–4 THz, the averaging THz EMI SE is 114.6 dB (Fig. 5c). In addition, in the reflection mode, when terahertz waves contact the Al mirror, electromagnetic waves were reflected and exhibited a strong terahertz signal. And when contact with the HMN composite films, the obtained electromagnetic signals undergo significant attenuation, which proves that HMN composite film can effectively absorb THz electromagnetic waves (Fig. 5d, e). Within the range of 0.4–2.8 THz, the peak of reflecting electromagnetic waves reflection loss (*R*_L_) has reached 39.7 dB at 0.6 THz, and the effective absorption bandwidth (*R*_L_ > 10 dB) is up to 2.1 THz (Fig. 5f). Furthermore, THz imaging technology is utilized to visualize the shielding and absorbing ability of the composite films for THz waves. The THz imaging suggests that the terahertz wave signal undergoes precipitous attenuation post-contact with the HMN composite films. The aforementioned results validate that the HMN composite films possess good shielding and absorbing ability for terahertz waves (Fig. 5g, h).

**Fig. 6:**
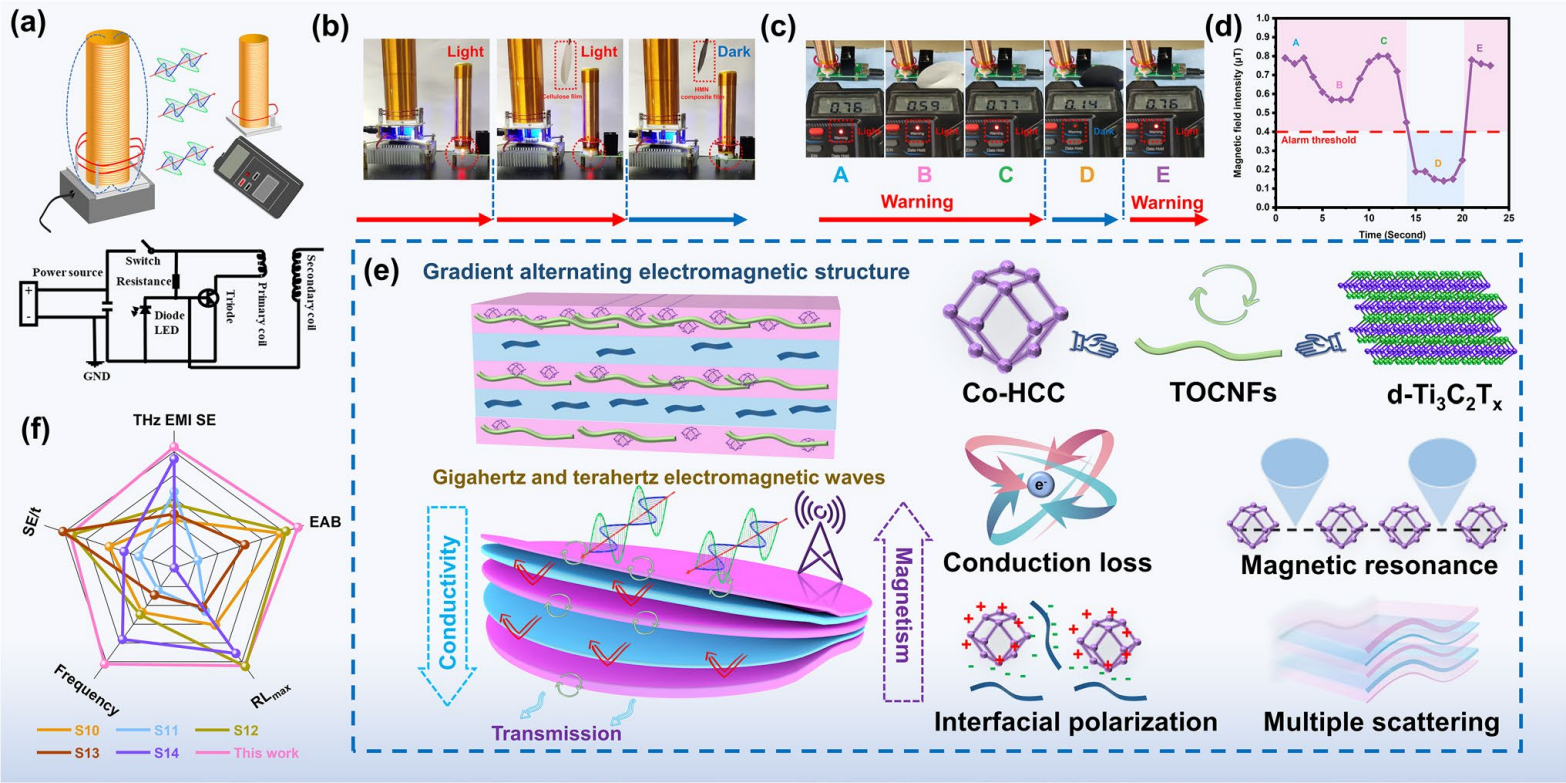
EMI simulated visually and EMI shielding mechanism. **a** Schematic diagram of the EMI simulated visually and Tesla coil circuit diagram. **b** Tesla coil experiment of HMN composite films. **c** Electromagnetic shielding detector EMI performance and **d** experiment of HMN composite films. **e** Schematic diagram of the EMI shielding mechanism of HMN composite films. **f** Comparison of the EMI SE with other materials (sample numbers listed in Table S2 in Supporting Information)

As displayed in Fig. 6a, the EMI applications of HMN composite films were simulated visually. The electromagnetic field engendered by the Tesla coil renders it capable of serving as energy to stimulate a secondary coil (Fig. 6b). The high-frequency electromagnetic field is conveyed to the secondary coil via the radiation of the Tesla coil. Upon receipt of high-frequency electromagnetic radiation from the secondary coil, its charge becomes activated, causing electrons to transition and emanate energy, which is subsequently emitted in the form of luminescence, thus lighting the small bulb. The HMN composite films can obstruct the radiation, causing the small bulb to extinguish. However, the pure TOCNFs films cannot obstruct the radiation. Hence, the small bulb is still lighting (Movie S1). In addition, it can also be seen from the electromagnetic radiation detector that when the HMN composite films are blocked between the Tesla coil and the detector, the electromagnetic radiation alarm sound disappears, and when the films are removed, the alarm sound rings again (Fig. 6c, d and Movie S2). The EMI shielding mechanism of HMN composite films is depicted in Fig. 6e. The EMI shielding performance generally depends on mobile carriers, electric/magnetic dipoles, and internal interfaces [55]. Conductive loss is primarily caused by the movement and jumping of free electrons in the conductive channel [56, 57]. The conductivity component plays a pivotal role in influencing the electromagnetic shielding capabilities through the generation of a larger number of free charges [46]. Additionally, the asymmetric distribution of charges within materials can lead to dipole polarization. Materials with asymmetric charge distribution form dipoles that interact with the magnetic field. This interaction leads to the loss of electromagnetic waves. Dipole polarization leads to better EMI shielding performance [58]. Furthermore, magnetic components can dissipate electromagnetic waves through the hysteresis loss, eddy current loss, domain wall resonance, ferromagnetic resonance, and natural resonance mechanisms [59, 60]. Under an alternating magnetic field, electrons will undergo spin motion and cause resonance between the magnetic filler and the magnetic field, resulting in the loss of electromagnetic waves [61]. When electromagnetic waves are incident from the initial layer of the HMN composite films, there will be losses resulting from scattering and absorption by the TOCNFs/Co-HCC with the mechanism of magnetic loss and dielectric loss. Furthermore, the highly graphitized out shell carbon framework present in Co-HCC also forms the conductive pathway and abundant heterogeneous interfaces, further enhancing the loss of electromagnetic waves [62]. When the residual electromagnetic wave passes through the initial layer to the next layer, part of the electromagnetic wave is lost due to interfacial polarization. Another part is reflected to the initial layer or converted into heat energy due to the conductivity loss of d-Ti_3_C_2_T_*x*_. The electromagnetic wave transmitted through the first and two layers will contact the third layer again and then be absorbed or reflected again. The remaining electromagnetic waves will re-enter the subsequent layer and continue to be dissipated. Notably, the gradient electromagnetic alternating structure effectively utilizes the respective advantages of electromagnetic components to enhance the loss ability of electromagnetic waves. Additionally, with the gradient positive sequence increase of d-Ti_3_C_2_T_*x*_ and gradient backward sequence increase content of TOCNFs/Co-HCC, more electromagnetic waves are absorbed in the front layers and reflected in the back layers, thus achieving the advantage of improving the EMI SE and realize the optimization of electromagnetic components. The intermittent and gradient conductive network is beneficial to improve the electromagnetic shielding performance of materials [63]. On the contrary, due to the disordered distribution of electromagnetic components, materials with random structures cannot form an effective electromagnetic wave loss network. Lack of an organized structure also leads to a loss of the ability to reflect and absorb the energy, resulting in a low level of electromagnetic shielding [64]. Hence, the design of the structure will produce gradient impedance matching, and the multi-layer structure introduced will cause extra electromagnetic energy loss at the corners generated by electromagnetic waves [65, 66]. The comparison of the EMI SE with other materials is displayed in Fig. 6f and Table S1.

### Photothermal Conversion Performances and Applicability of HMN Composite Films

**Fig. 7:**
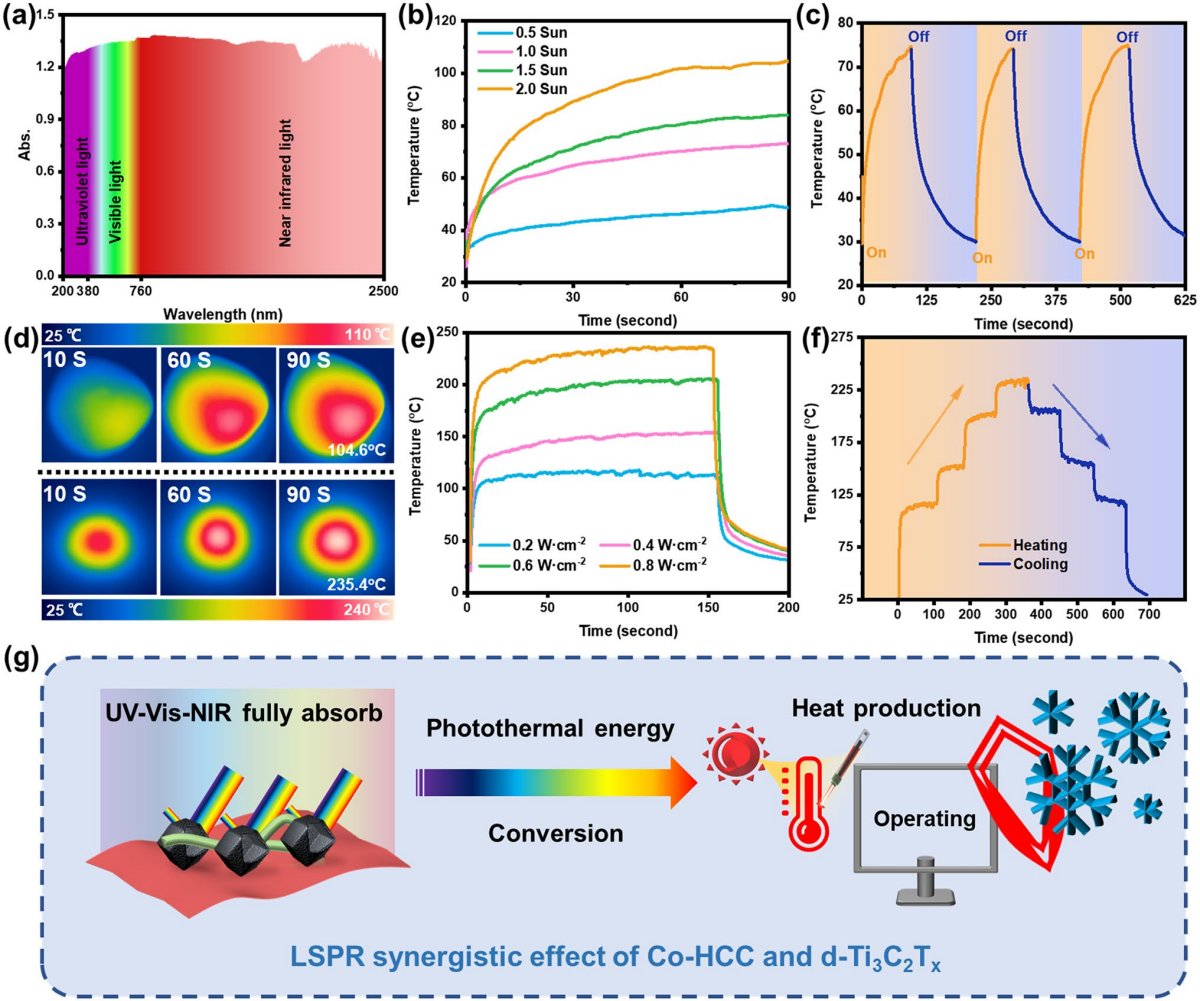
Photothermal conversion performances of HMN composite films. **a** UV–Vis–NIR absorbance spectrum of HMN composite films. **b** Surface temperature curves for the HMN composite films under different solar intensity. **c** Solar-heating performance of HMN composite films in 3 cycles with an applied solar intensity of 1.0 Sun. **d** Infrared thermographic photographs of HMN composite films under 2.0 Sun (upper) and NIR irradiation (lower) under 0.8 W cm^−2^ at different time intervals. **e** Surface temperature curves for the HMN composite films under different 808 NIR power densities. **f** Temperature evolution of HMN composite films at different 808 NIR laser power densities. **g** Schematic diagram of HMN composite films irradiated by simulant photothermal

Deriving from the local surface plasmon resonance (LSPR) effect for solar radiation, MXene and MOFs have efficient light-to-heat conversion capability [67, 68]. MXene/MOFs functional materials have been widely used in photothermal conversion devices. The light absorption performance of materials is one of the important factors affecting photothermal conversion. As displayed in Fig. 7a, the absorption spectra of HMN composite films at all solar wavelengths were measured by UV–Vis-NIR spectrophotometer. The absorption capacity of HMN composite film shows a high level in the whole wavelength range, which proves its strong absorption capacity. As shown in Fig. 7b, d, HMN composite films exhibited high solar thermal conversion performance. Under the irradiation of 0.5, 1, 1.5, and 2.0 Sun (1 Sun = 1 KW m^−2^), the surface temperature of HMN-5L-57.1% rapidly reached to 48.6, 73.2, 84.1, and 104.6 °C, respectively. Besides, the solar response ability and cyclic response ability are displayed in Fig. 7c. After the simulated sunlight was turned off, the surface temperature of HMN composite films rapidly decreased. Even after repeated three cycles, it still maintains good solar conversion ability. Furthermore, given that the NIR laser possesses a higher power density, it can be used to quickly raise the photothermal material to a higher temperature in a shorter time. The NIR laser irradiation thermal conversion performance of HMN composite films was investigated. As shown in Fig. 7d, e, the HMN-5L-57.1% displayed obvious temperature gradient changes with different NIR laser irradiation intensity proportions from 0.2 to 0.8 W cm^−2^. The highest surface temperature can reach 235.4 °C under 0.8 W cm^−2^. Furthermore, through the alternating state of temperature rise/fall and repeated light on/off with different radiation intensity, the stability of NIR laser irradiation thermal conversion of HMN-5L-57.1% was confirmed (Fig. 7f). Temperature evolution processes of HMN-5L-57.1% were stable during different NIR laser power densities irradiation. Besides, the highest surface temperature and temperature rise rate curve of HMN composite films are basically consistent under 20 consecutive cycles of on/off and long-term illumination, and it has more cycles of on/off ability and continuous heating time than previously reports (Figs. S21, S22 and Table S3). The solar-to-thermal conversion efficiency of the HMN composite films was measured. Due to ultra-high light absorption rate and low near-infrared emissivity, the photothermal conversion efficiency of HMN composite films can reach 66.2% (Figs. S23 and S24). CNT derived from the surface of Co-HCC endows nanoparticles with a rougher surface, which is beneficial to generate more hot spots, thus improving the photothermal conversion ability of materials. In addition, LSPR is further promoted in the hollow structure and heat loss can be reduced. The hierarchical structure and the LSPR synergistic effect of d-siTi_3_C_2_T_*x*_ and Co-HCC endow HMN composite films with excellent photothermal conversion on ability (Fig. 7g) [69].

With the miniaturization and precision of electronic equipment, integrated multifunctional electromagnetic shielding materials need to be light, thin, and flexible. As shown in Fig. S25a, HMN composite films have the characteristics of low density (placed on dandelion fluff without collapse), ultra-thin (the thickness is only ~ 0.2 mm), and flexible (bent on the glass rod without crack). In addition, through cutting, HMN composite film can be cut into any shape to meet a variety of personalized needs (Fig. S25b). Besides, the mechanical performance of HMN composite films was further investigated. The good flexibility of HMN composite films can endow it with the property that it can withstand bending at different angles without breaking (Fig. S26). The typical tensile stress–strain curves are shown in Fig. S27. The ultimate strengths of the d-Ti_3_C_2_T_*x*_, TOCNF, and HMN composite films are about 2.7, 14.3, and 18.7 MPa, respectively. Additionally, the tensile strain percentage of the d-Ti_3_C_2_T_x_, TOCNF, and HMN composite films is about 1.0%, 5.5%, and 4.8%. The mechanical properties are helpful to improve the adaptability of electromagnetic shielding materials in various application scenarios [70]. Interestingly, because Co-HCC nanoparticles have high magnetism, they can be easily attracted by magnets without falling off. This is beneficial to be attached to the surface of devices or implanted in magnetic fabrics in the future (Fig. S28 and Movie S3). Moreover, due to the design of the multi-layer structure, the d-Ti_3_C_2_T_*x*_ layer is protected by the outer TOCNFs/Co-HCC layer, and the HMN composite films can be kept intact in the 180-W ultrasonic process, while the pure d-Ti_3_C_2_T_*x*_ films were quickly dissolved in water within 30 s. Hydrogen bonding and physical entanglement between TOCNFs endow HMN composite films with moisture resistance (Fig. S29) [71].

## Conclusions

In summary, we prepared hollow MOFs/layered MXene/nanocellulose composite films with alternating electromagnetic structures via macro/micro–electromagnetic structural design. The HMN composite films are applied for ultra-wideband giga/terahertz electromagnetic shielding and photothermal conversion. The HMN composite films displayed excellent EMI shielding effectiveness (EMI SE) performance in the GHz frequency (average 66.8 dB at Ka-band) and THz frequency (average 114.6 dB at 0.1–4.0 THz). Besides, the HMN composite films also exhibit a high reflection loss of 39.7 dB at 0.7 THz with an effective absorption bandwidth up to 2.1 THz. Moreover, the electromagnetic shielding application of HMN composite film is proved by simulating and visualizing the electromagnetic shielding application. Furthermore, HMN composite films show remarkable solar/laser photothermal conversion performance, which can reach 104.6 °C under 2.0 Sun and 235.4 °C under 0.8 W cm^−2^, respectively. Therefore, the HMN composite films with structural design will be highly promising for a promising candidate for advanced EMI devices for future 6G electronic communication and the protection of electronic equipment in cold environments.

## Supplementary Information

Below is the link to the electronic supplementary material.

Supplementary file1 (MP4 1248 KB)

Supplementary file2 (MP4 1518 KB)

Supplementary file3 (MP4 619 KB)

Supplementary file4 (PDF 1495 KB)
