# Supplementary material for: Hollow Metal–Organic Framework/MXene/Nanocellulose Composite Films for Giga/Terahertz Electromagnetic Shielding and Photothermal Conversion
Source: Nanomicro Lett. 2024 Apr 8;16:169. doi: 10.1007/s40820-024-01386-5 (PMC11001847; doi:10.1007/s40820-024-01386-5)
Supplement: Supplementary file 4 — Supplementary file4 (PDF 1495 KB) [file 40820_2024_1386_MOESM4_ESM.pdf]

Support Information for

Hollow Metal-Organic Framework/MXene/Nanocellulose Composite Films for Giga/Terahertz Electromagnetic Shielding and Photothermal Conversion

Tian Mai [1], Lei Chen [1], Pei-Lin Wang [1], Qi Liu [1], Ming-Guo Ma [1, 2, *]

[1] Research Center of Biomass Clean Utilization, MOE Engineering Research Center of Forestry Biomass Materials and Bioenergy, Beijing Key Laboratory of Lignocellulosic Chemistry, College of Materials Science and Technology, Beijing Forestry University, Beijing 100083, P. R. China

[2] State Silica-based Materials Laboratory of Anhui Province, Bengbu 233000, P. R. China

*Corresponding author. E-mail: mg_ma@bjfu.edu.cn (Ming-Guo Ma)

Supplementary Figures, Tables and Movies

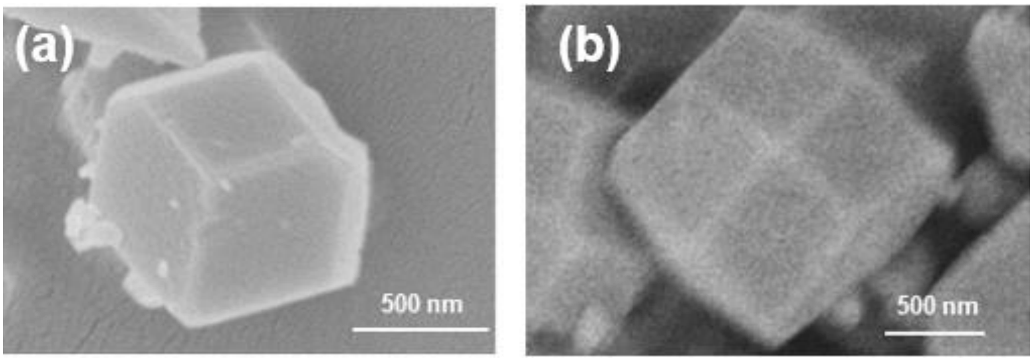

Fig. S1 SEM images of **a** ZIF-8 and **b** ZIF-8@ZIF-67

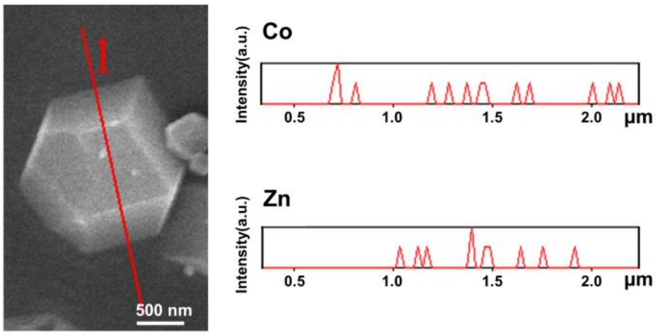

Fig. S2 EDS line scan image of ZIF-8@ZIF-67

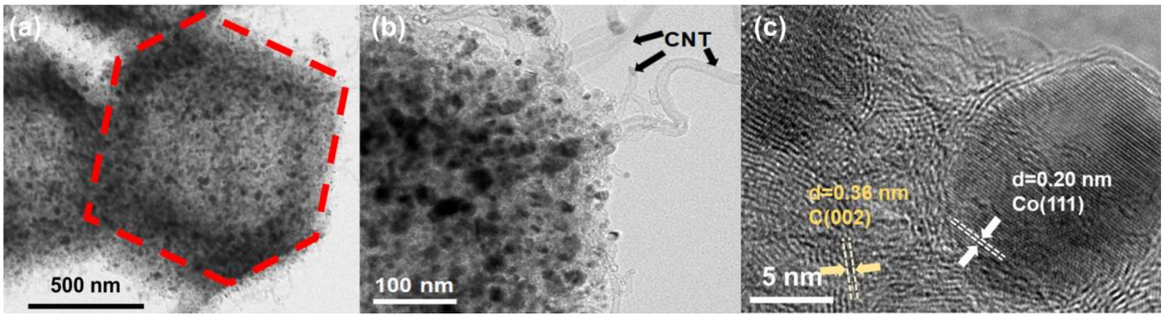

Fig. S3 TEM images of **a** Co-HCC, **b** CNT of Co-HCC, and **c** the lattice fringe of Co-HCC

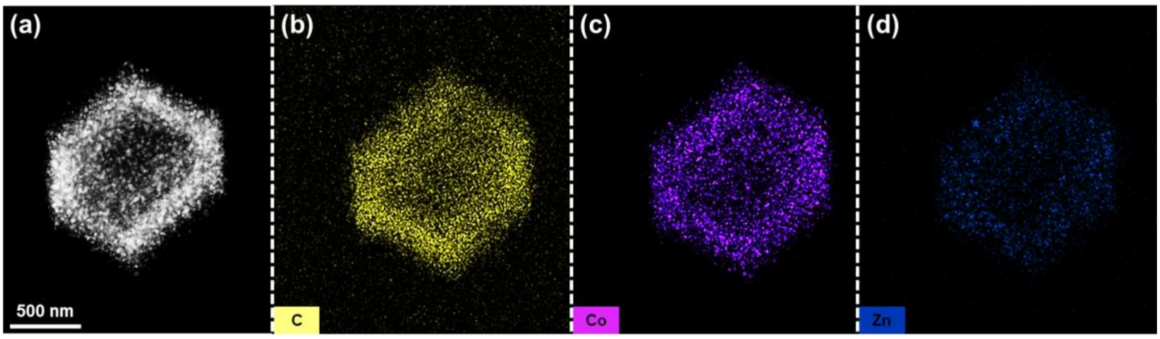

Fig. S4 EDS mapping images of Co-HCC

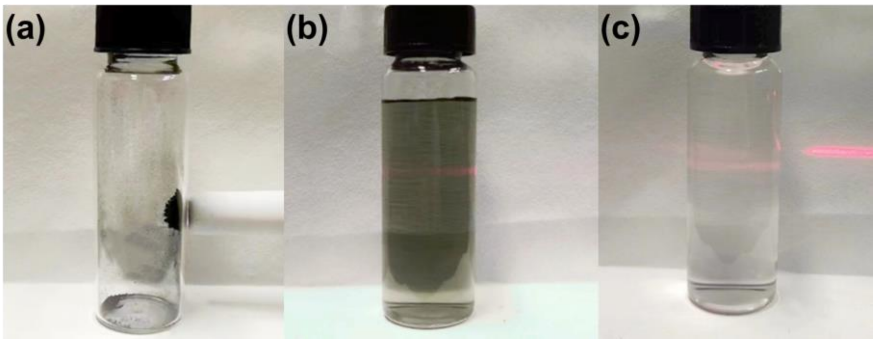

Fig. S5 a Magnetism of Co-HCC. The Tyndall effect of **b** d-$Ti_3C_2T_x$ and **c** TOCNFs, respectively

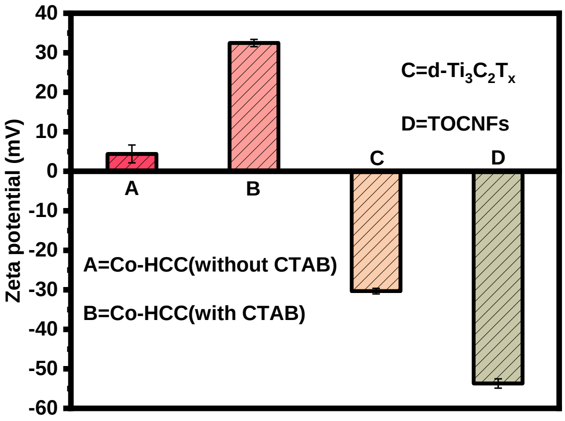

Fig. S6 Zeta-potential measurement of TOCNFs, d-$Ti_3C_2T_x$, and Co-HCC before and after CTAB modification (pH = 7, in water)

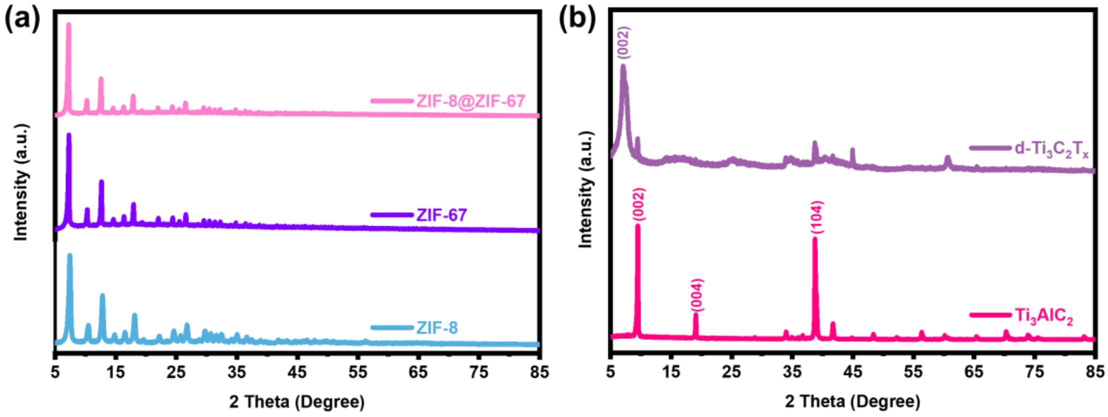

Fig. S7 a XRD patterns of ZIF-8@ZIF-67, ZIF-67, and ZIF-8, respectively. **b** The XRD patterns of Ti_3AlC_2 and d-$Ti_3C_2T_x$

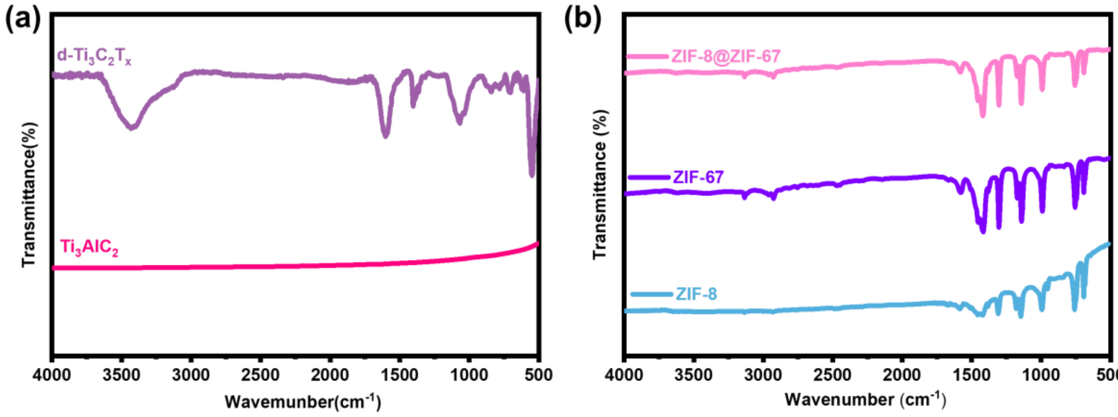

Fig. S8 FTIR spectra of **a** Ti_3AlC_2 and d-$Ti_3C_2T_x$, **b** ZIF-8@ZIF-67, ZIF-67, and ZIF-8, respectively

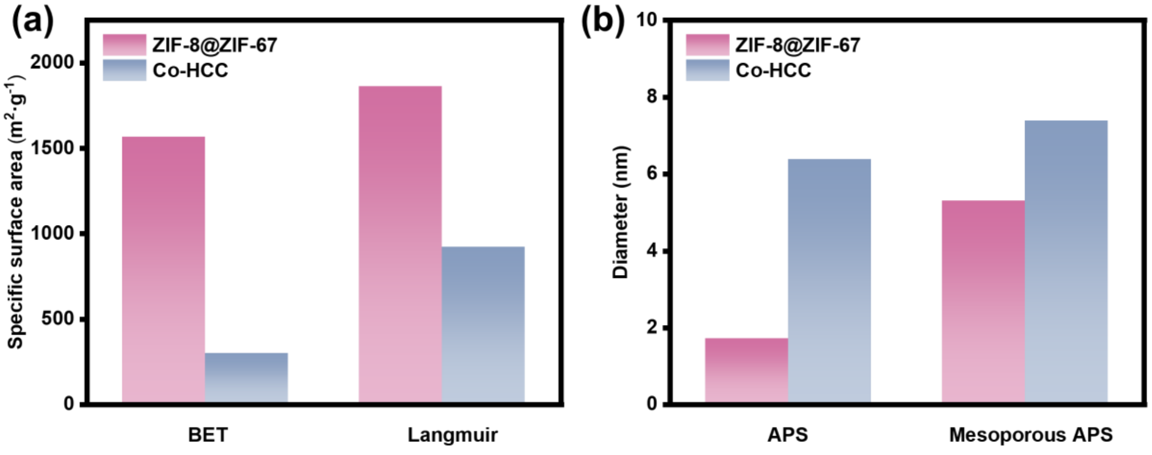

Fig. S9 a Specific surface area and **b** pore diameter of ZIF-8@ZIF-67 and Co-HCC

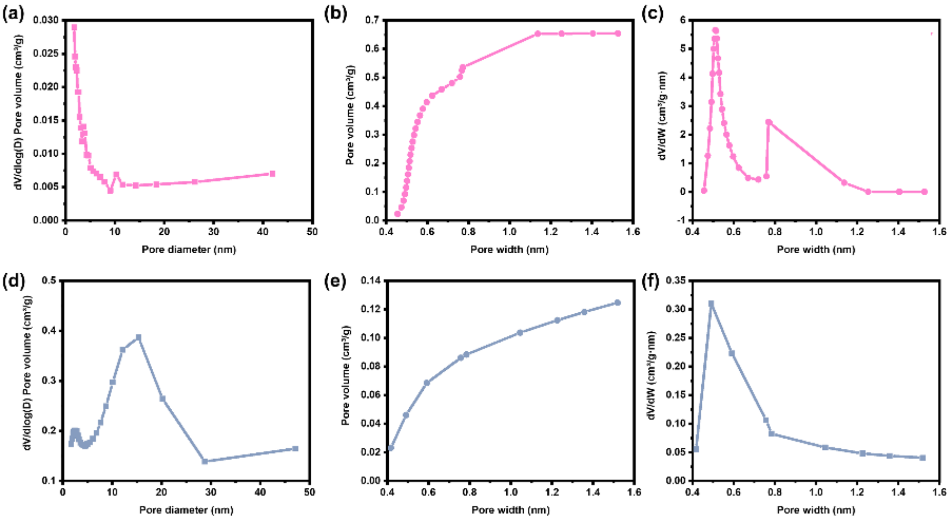

Fig. S10 a, d BJH adsorption dV/dlog(D) pore volume, **b, e** Horvath-Kawazoe cumulative pore volume Plot, and **c, f** Horvath-Kawazoe differential pore volume plot of ZIF-8@ZIF-67 and Co-HCC, respectively

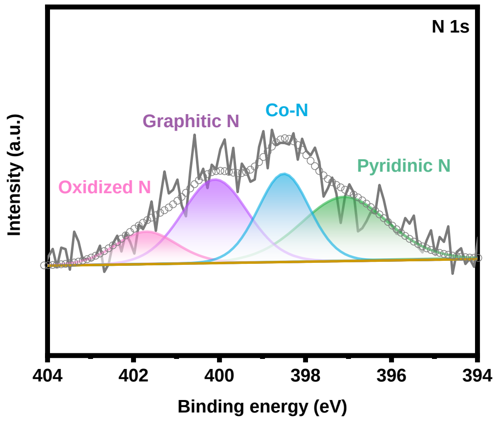

Fig. S11 XPS fine spectra of N 1s of Co-HCC

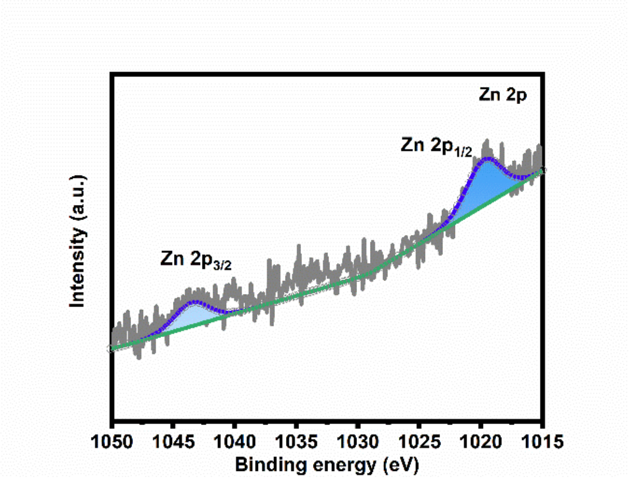

Fig. S12 XPS fine spectra of Zn 2p of Co-HCC

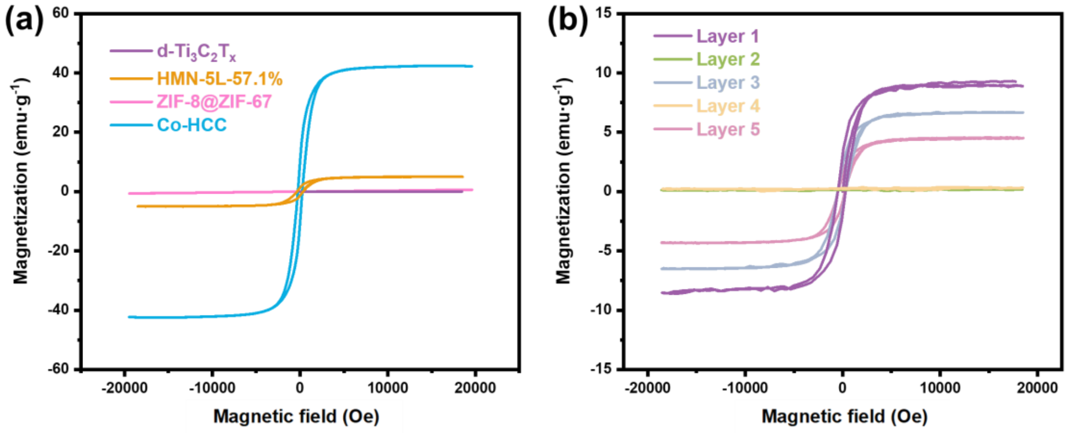

Fig. S13 The magnetic hysteresis loop of **a** d-$Ti_3C_2T_x$, HMN-5L-57.1%, ZIF-8@ZIF-67, Co-HCC, and **b** each layer of HMN-5L-57.1%, respectively

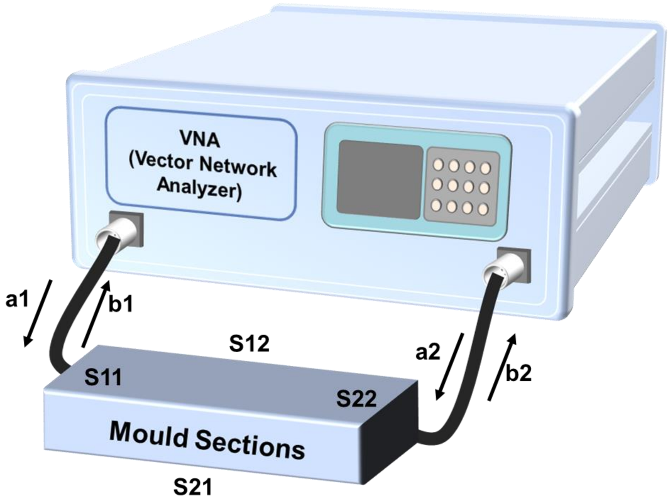

Fig. S14 Schematic diagram of vector network analyzer in GHz electromagnetic shielding test

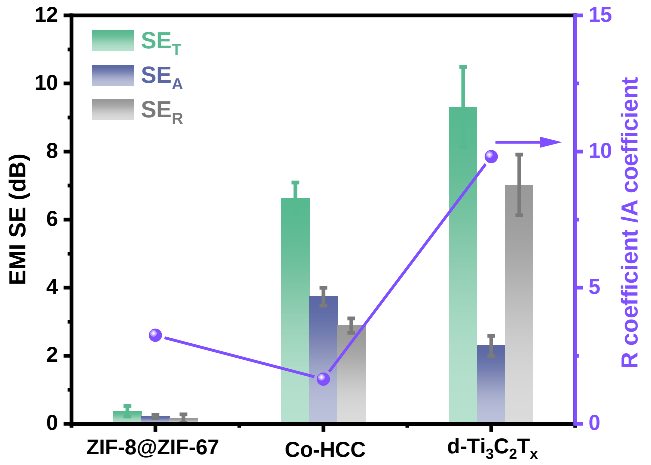

Fig. S15 EMI SE and R/A coefficient of ZIF-8@ZIF-67, Co-HCC, and d-$Ti_3C_2T_x$

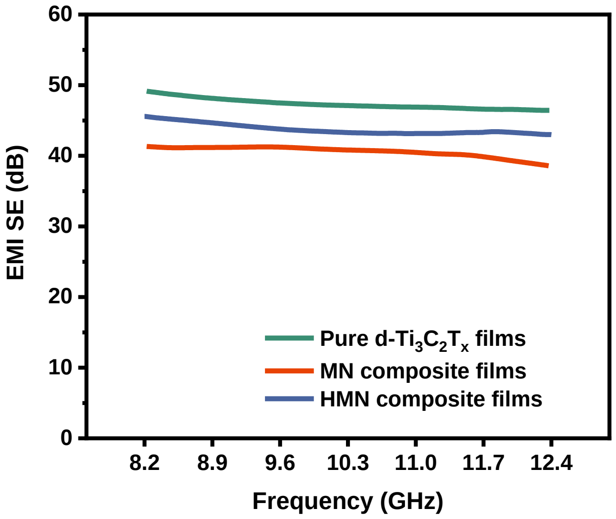

Fig. S16 EMI SE of pure d-$Ti_3C_2T_x$ films, MN composite films, and HMN composite films

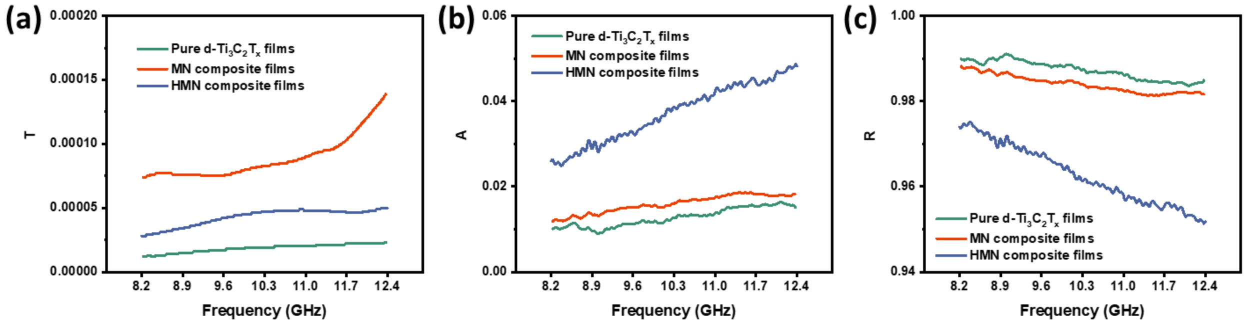

Fig. S17 T, A, and R coefficient of pure d-$Ti_3C_2T_x$ films, MN composite films, and HMN composite films

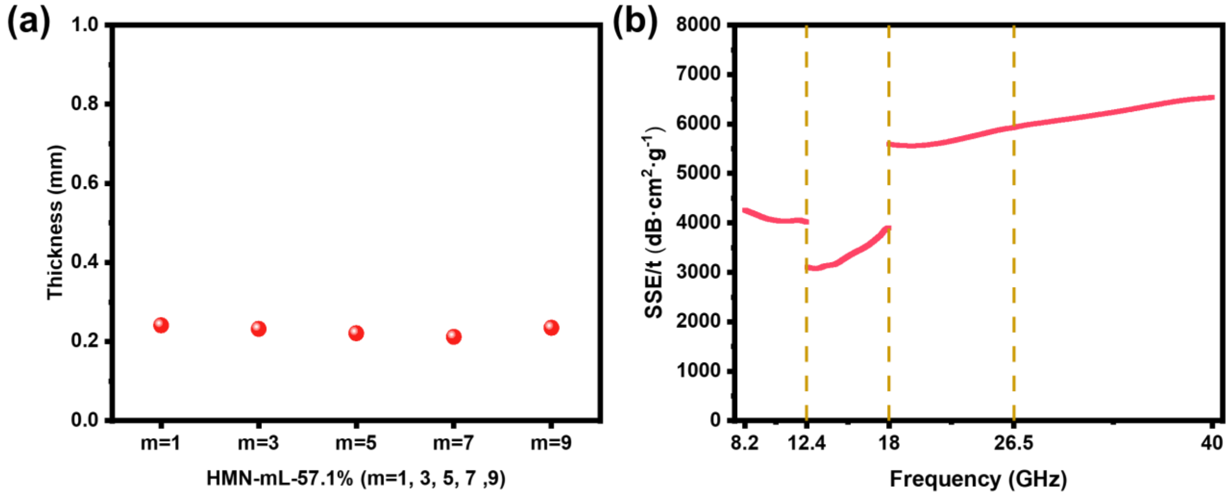

Fig. S18 a The thickness and **b** SSE/t of HMN composite films

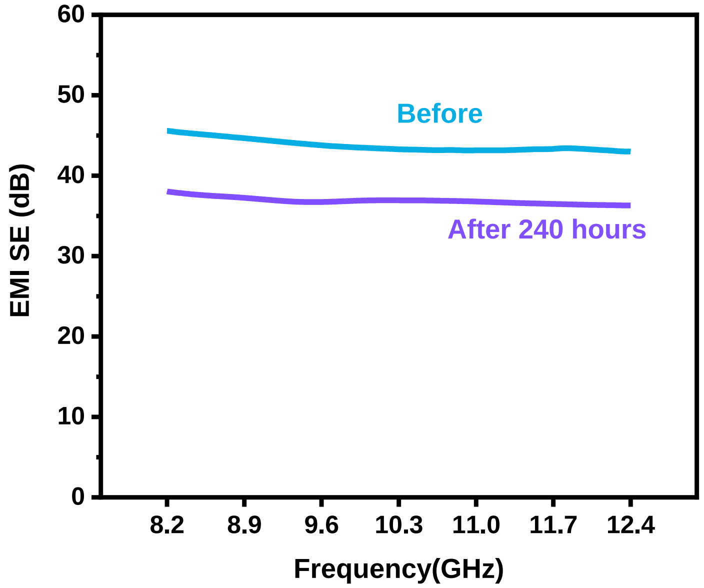

Fig. S19 EMI SE of HMN composite films before and after stored at room temperature for 240 hours

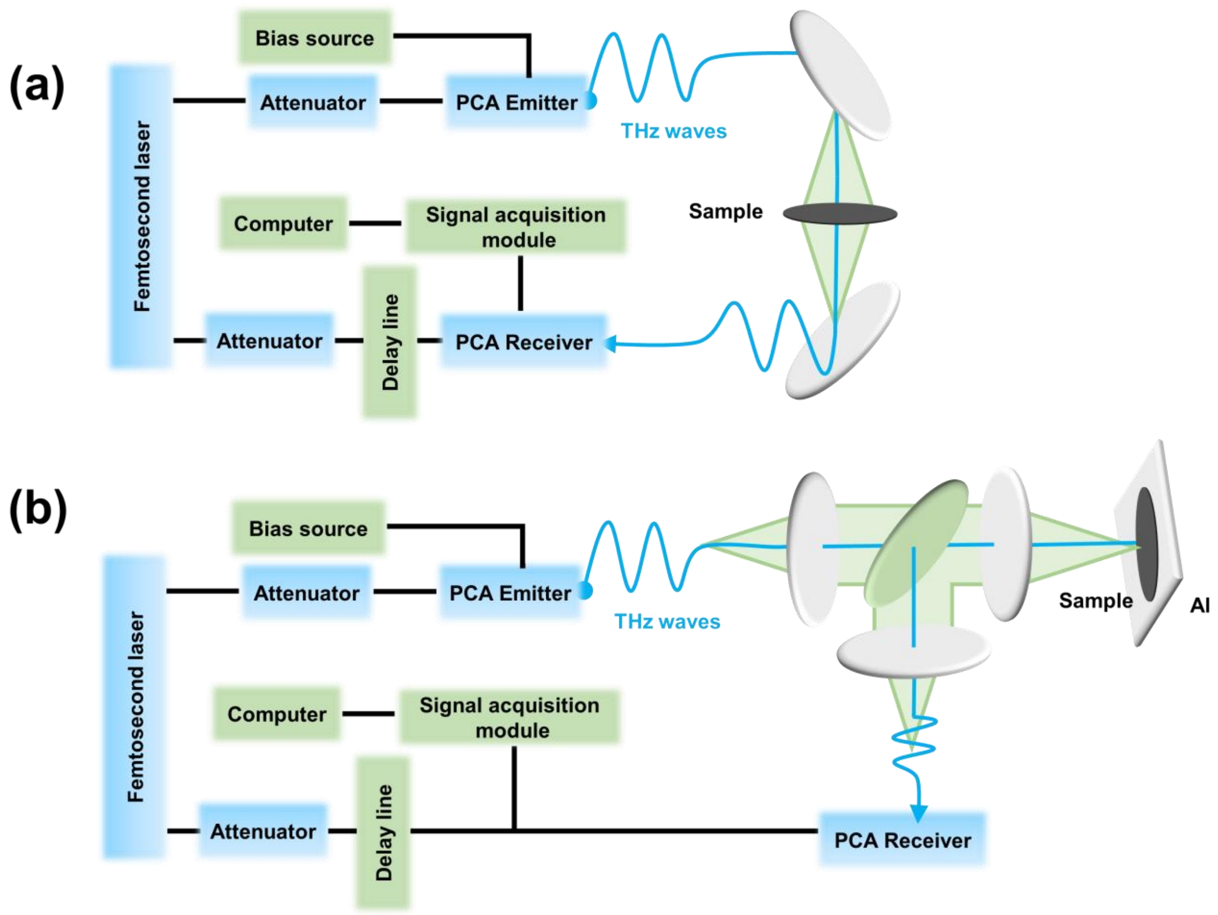

Fig. S20 a Transmission mode system and **b** reflection mode system of time-domain THz spectroscopy (PCA= Photoconductive antenna)

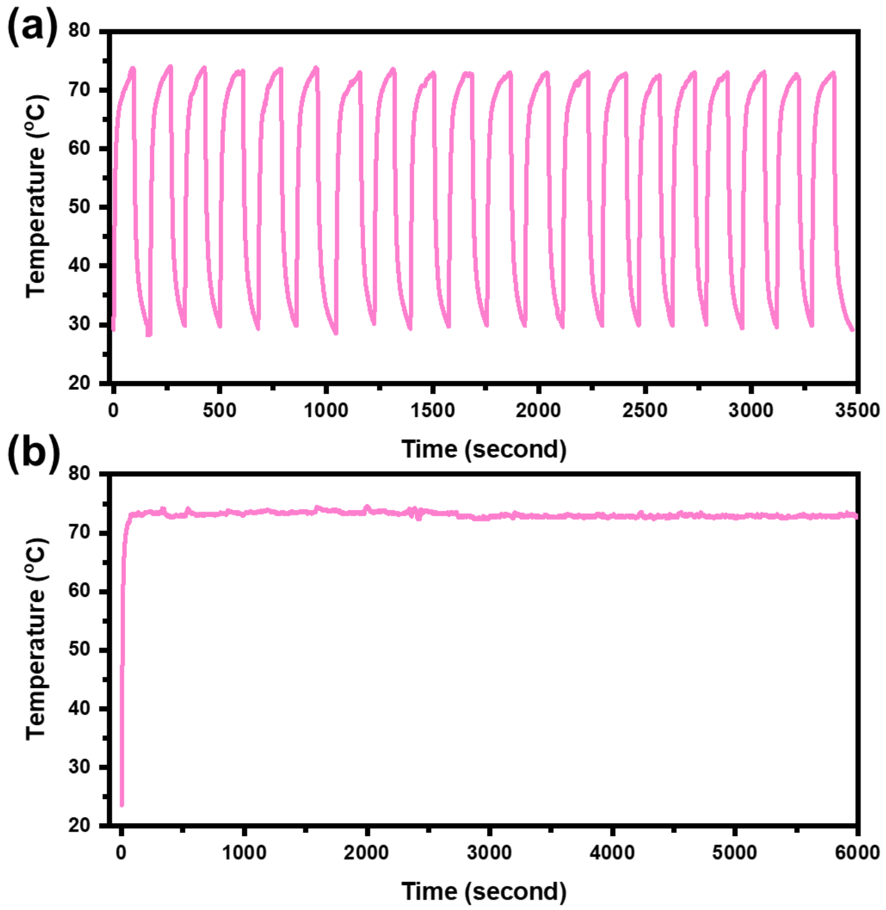

Fig. S21 Solar-heating performance of HMN composite films in 20 cycles **a** and 6000 s continuous illumination **b** with an applied solar intensity of 1.0 Sun

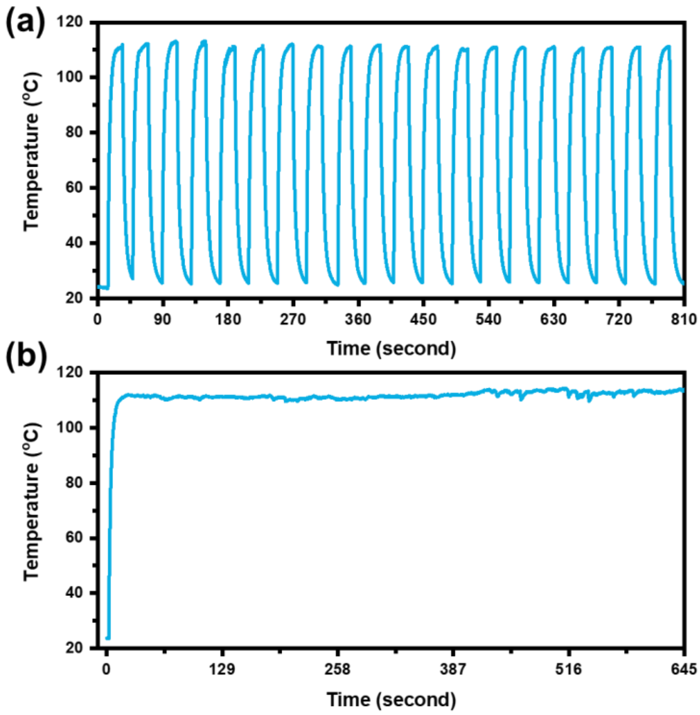

Fig. S22 NIR irradiation photothermal performance of HMN composite films in 20 cycles **a** and 645 s continuous illumination **b** with an applied solar intensity of 0.2 W·cm^{-2}

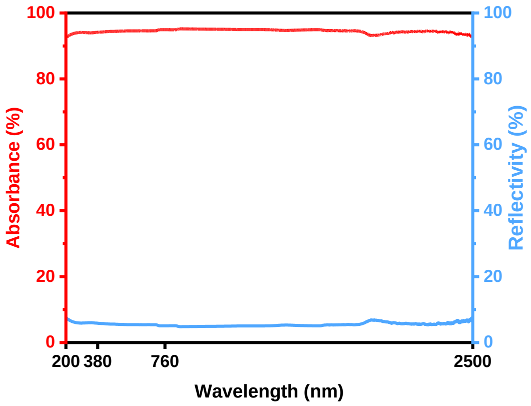

Fig. S23 Light absorbance and reflectivity of HMN composite films

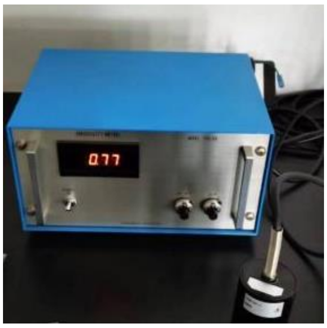

Fig. S24 IR emissivity of HMN composite films

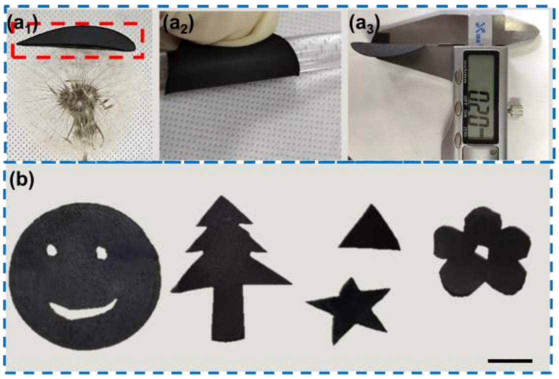

Fig. S25 **a** Optical images show the characteristics of low density, flexible, and ultrathin for HMN composite films respectively. **b** Cutability of HMN composite films. (The scale bar is 1 cm)

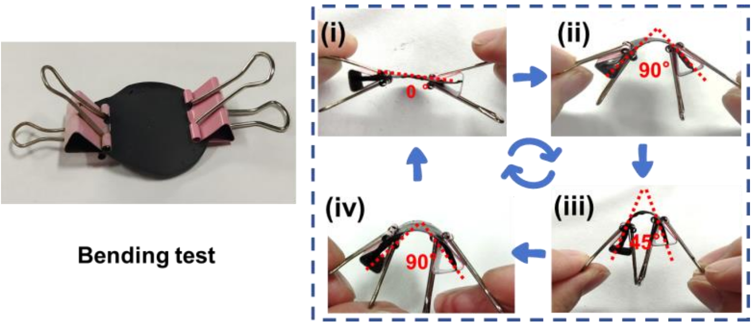

Fig. S26 Bending test of HMN composite films

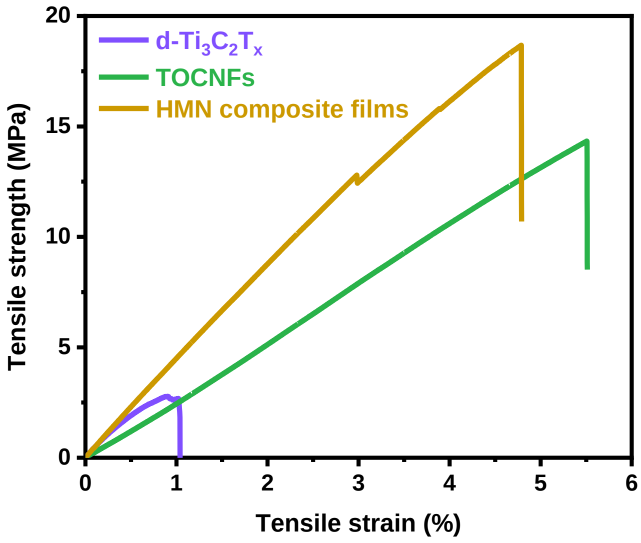

Fig. S27 Stress-strain curve of HMN composite films, TOCNFs, and d-$Ti_3C_2T_x$

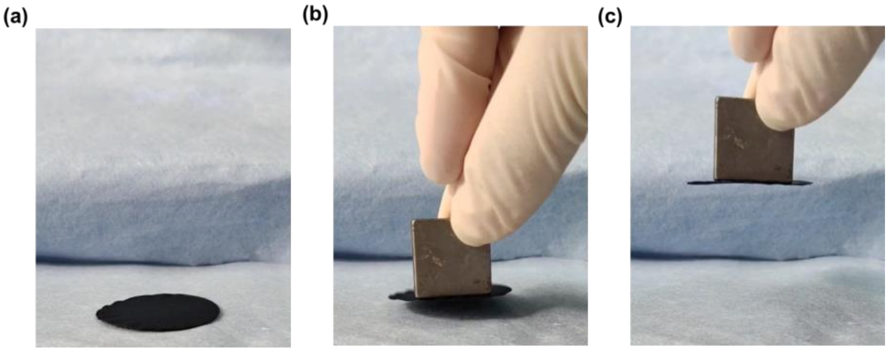

Fig. S28 Process of attracting HMN composite films by magnets

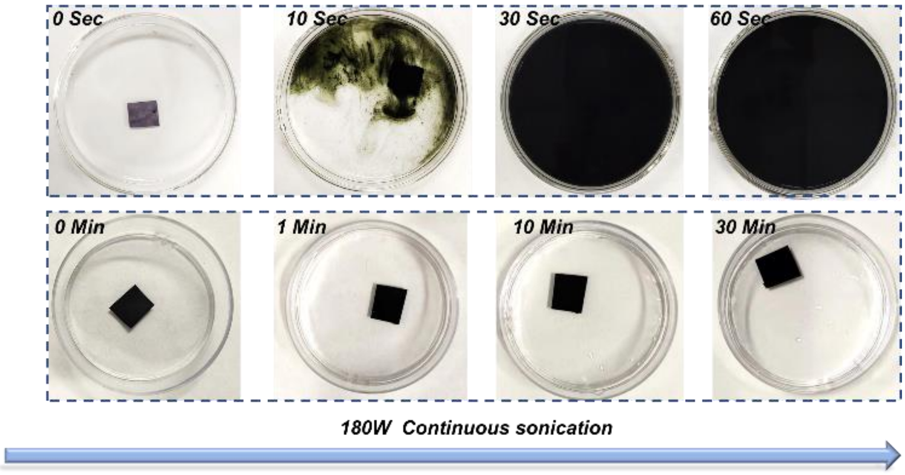

Fig. S29 Ultrasonic stability of pure d-$Ti_3C_2T_x$ films (Upper) and HMN composite films (Lower)

Table S1 Comparison of comprehensive GHz EMI performance between HMN composite film and other materials

**Materials**	X band	Ku-band	K-band	Ka-band	**Refs.**
**TPU/graphene films**	~20 dB	~21 dB	~22 dB	~25 dB	[S1]
**TPU/15AC@4M−SW**	35.7 dB	38.9 dB	-	-	[S2]
**MWCNTs/epoxy**	-	-	-	41 dB	[S3]
**DMD-based Ag film**	~50 dB	~55 dB	~50 dB	~55 dB	[S4]
**$Ti_3C_2T_x$/CIs**	31.9 dB	-	-	-	[S5]
**ITO nano-branch**	-	-	21.09 dB	17.81 dB	[S6]
**PVDF/GnP**	-	-	-	37.4 dB	[S7]
**TPU/CAPP/MSCF**	43.8 dB	32.0 dB	-	-	[S8]
**GO@dough**	33.95 dB	-	-	-	[S9]
**HMN-5L-57.1%**	43.7 dB	36 dB	60.9 dB	66.8 dB	This work

Table S2 Comparison of comprehensive THz EMI performance between HMN composite film and other materials

**Materials**	**EMI SE (dB)**	**Frequency (THz)**	**SE/t (dB·μm)**	**RL_{max} (dB)**	**EAB (THz)**	**Refs.**
**MXene/PAA**	45.3	0.2–2.0	0.35	23.2	1.8	[S10]
**rGO papers**	72.1	0.1–1.0	0.19	17.6	0.4	[S11]
**MXene/PI**	60.3	0.1–2.0	0.55	~40	0.5	[S12]
**Zn^{2+}/MXene**	51	0.2–2.0	0.6	~16	1.1	[S13]
**Fe_3GeTe_2**	103.2	0.1–3.0	0.27	34.7	0.0029	[S14]
**CS sponge**	49	0.25–3.5	0.006125	47	3.25	[S15]
**Borophene**	50	0.1–2.7	-	35	2.6	[S16]
**CNFs-Fe_3O_4**	60	0.1–1.2	0.11	-	-	[S17]
**HMN-5L-57.1%**	114.6	0.1–4.0	0.56	39.7	2.1	**This work**

Table S3 Comparison of the stability of the photothermal conversion between HMN composite film and other materials

Materials	Cycles (Solar)	Time (Solar)	Cycles (NIR laser)	Time (NIR laser)	Refs.
**PDMS@WSM**	10	1000 s	-	-	[S18]
**ANF/MXene-PEDOT:PSS**	50	4200 s	-	-	[S19]
**CNF/MoS_2**	-	-	15	~180 s	[S20]
**ANF-derived carbon**	11	4800 s	-	-	[S21]
**$Ti_3C_2T_x$/BC**	-	-	8	1200 s	[S22]
**TPU/MXene**	5	3600 s	-	-	[S23]
**HMN-5L-57.1%**	20	6000 s	20	645 s	**This work**

Table S4 Mass percentage of each component of samples

Constituent	Co-HCC (wt.%)	TOCNFs (wt.%)	d-$Ti_3C_2T_x$ (wt%)
HMN-1L-57.1 wt.%	14.3	28.6	57.1
HMN-3L-57.1 wt.%	14.3	28.6	57.1
HMN-5L-57.1 wt.%	14.3	28.6	57.1
HMN-5L-50.0 wt.%	16.7	33.3	50.0
HMN-5L-40.0 wt.%	20.0	40.0	40.0
HMN-5L-25.0 wt.%	25.0	50.0	25.0
HMN-5L-0.0 wt.%	33.3	66.7	0
HMN-7L-57.1 wt.%	14.3	28.6	57.1
HMN-9L-57.1 wt.%	14.3	28.6	57.1

Table S5 Content of each component of samples

Constituent	Co-HCC (mg)	TOCNFs(mg)	d-$Ti_3C_2T_x$ (mg)
HMN-1L-57.1 wt.%	30	60	120
HMN-3L-57.1 wt.%	30	60	120
HMN-5L-57.1 wt.%	30	60	120
HMN-5L-50.0 wt.%	30	60	90
HMN-5L-40.0 wt.%	30	60	60
HMN-5L-25.0 wt.%	30	60	30
HMN-5L-0.0 wt.%	30	60	0

HMN-7L-57.1 wt.%	30	60	120
HMN-9L-57.1 wt.%	30	60	120

Table S6 Mass percentage of each layer component of HMN-5L-57.1 wt%

Constituent	Co-HCC (wt.%)	TOCNFs (wt.%)	d-$Ti_3C_2T_x$ (wt%)
**Layer 1**	7.1	14.3	0
**Layer 2**	0	0	19.0
**Layer 3**	4.8	9.5	0
**Layer 4**	0	0	38.1
**Layer 5**	2.4	4.8	0

Table S7 Content of each layer component of HMN-5L-57.1 wt%

Constituent	Co-HCC (mg)	TOCNFs (mg)	d-$Ti_3C_2T_x$ (mg)
**Layer 1**	15	30	0
**Layer 2**	0	0	40
**Layer 3**	10	20	0
**Layer 4**	0	0	80
**Layer 5**	5	10	0

Supplementary Movie S1

The variation of LED brightness in Tesla coil experiment with and without HMN composite films.

Supplementary Movie S2

The alarm of the electromagnetic radiation detector in Tesla coil experiment with and without HMN composite films.

Supplementary Movie S3

HMN composite films are attracted by magnets.

Supplementary References

[S1] B. Shen, Y. Li, D. Yi, W. Zhai, X. Wei et al., Strong flexible polymer/graphene composite films with 3D saw-tooth folding for enhanced and tunable electromagnetic shielding. Carbon **113**, 55–62 (2017). https://doi.org/10.1016/j.carbon.2016.11.034

[S2] K. Chen, H. Wang, Y. Shi, M. Liu, Y. Feng et al., Realizing balanced flame retardancy and electromagnetic interference shielding in hierarchical elastomer nanocomposites. J. Colloid. Interface Sci. **653**, 634–642 (2024). https://doi.org/10.1016/j.jcis.2023.09.112

[S3] Z. Wang, G.D. Wei, G.L. Zhao, Enhanced electromagnetic wave shielding effectiveness

of Fe doped carbon nanotubes/epoxy composites. Appl. Phys. Lett. **103**(18), 183109 (2013). https://doi.org/10.1063/1.4828356

[S4] H. Wang, C. Ji, C. Zhang, Y. Zhang, Z. Zhang et al., Highly transparent and broadband electromagnetic interference shielding based on ultrathin doped Ag and conducting oxides hybrid film structures. ACS Appl. Mater. Interfaces **11**(12), 11782–11791 (2019). https://doi.org/10.1021/acsami.9b00716

[S5] G. Yang, X. Yao, Y. Li, Y. Hu, X. Chen et al., $Ti_3C_2T_x$ MXene nanosheet-based hybrid films for enhanced wave absorption-dominated electromagnetic interference shielding. ACS Appl. Nano Mater. **6**(20), 19378–19391 (2023). https://doi.org/10.1021/acsanm.3c03932

[S6] Y. Kim, N. Kim, S.H. Lee, S.K. Hyeong, J.H. Lee et al., Enhanced ultra high frequency emi shielding with controlled ITO nano-branch width via different TIN material types. Nanoscale **15**(33), 13635–13644 (2023). https://doi.org/10.1039/d3nr03153e

[S7] B. Zhao, C. Zhao, M. Hamidinejad, C. Wang, R. Li et al., Incorporating a microcellular structure into PVDF/graphene–nanoplatelet composites to tune their electrical conductivity and electromagnetic interference shielding properties. J. Mater. Chem. C **6**(38), 10292–10300 (2018). https://doi.org/10.1039/c8tc03714k

[S8] K.X. Chen, M. Liu, Y.Q. Shi, H.R. Wang, L.B. Fu et al., Multi-hierarchical flexible composites towards superior fire safety and electromagnetic interference shielding. Nano Res. **15**(10), 9531–9543 (2022). https://doi.org/10.1007/s12274-022-4883-6

[S9] Z.L. Liu, G.H. Wang, P.Y. Li, F.H. Xue, Z. Chen et al., Gradient in-plane oriented porous carbon inspired by fabrication of toasts for elegant emi shielding performance. Carbon **207**, 136–143 (2023). https://doi.org/10.1016/j.carbon.2023.03.013

[S10] Y. Zhu, J. Liu, T. Guo, J.J. Wang, X. Tang et al., Multifunctional $Ti_3C_2T_x$ MXene composite hydrogels with strain sensitivity toward absorption-dominated electromagnetic-interference shielding. ACS Nano **15**(1), 1465–1474 (2021). https://doi.org/10.1021/acsnano.0c08830

[S11] S.H. Dong, Q.W. Shi, W.X. Huang, L.L. Jiang, Y. Cai, Flexible reduced graphene oxide paper with excellent electromagnetic interference shielding for terahertz wave. J. Mater. Sci. Mater. Electron. **29**, 17245–17253 (2018). https://doi.org/10.1007/s10854-018-9818-1

[S12] V.C.S. Theja, D.S. Assi, H. Huang, R.S. Alsulami, B.J. Chen et al., 3D architectural MXene-based composite films for stealth terahertz electromagnetic interference shielding performance. Adv. Mater. Interfaces **10**(36), 2300440 (2023). https://doi.org/10.1002/admi.202300440

[S13] Z. Lin, J. Liu, W. Peng, Y. Zhu, Y. Zhao et al., Highly stable 3D $Ti_3C_2T_x$ MXene-based foam architectures toward high-performance terahertz radiation shielding. ACS Nano **14**(2), 2109–2117 (2020). https://doi.org/10.1021/acsnano.9b08832

[S14] G. Li, S. Ma, Z. Li, Y. Zhang, J. Diao et al., High-quality ferromagnet Fe_3GeTe_2 for high-efficiency electromagnetic wave absorption and shielding with wideband radar cross section reduction. ACS Nano **16**(5), 7861–7879 (2022). https://doi.org/10.1021/acsnano.2c00512

[S15] J. Yang, S. Meng, Y.C. Li, Y.X. Ye, J. Tan et al., Low-cost, flexible and broadband terahertz absorber based on annealed carbonized sponge. J. Mater. Sci. Mater. Electron. **33**, 379–387 (2022). https://doi.org/10.1007/s10854-021-07307-1

[S16] Z. Zhang, M. Yang, Y. Zhang, M. Zhou, Research and application of terahertz response mechanism of few-layer borophene. Nanomaterials **12**(15), 2702 (2022). https://doi.org/10.3390/nano12152702

[S17] N. Arooj, T.M. Khan, M. Mumtaz, A. Rehman, I. Ahmad et al., Polymeric Fe_3O_4 nanoparticle/carbon nanofiber hybrid nanocomposite coatings for improved terahertz shielding. ACS Appl. Nano Mater. **6**(7), 5264–5273 (2023). https://doi.org/10.1021/acsanm.2c05313

[S18] P.L. Wang, C. Ma, Q. Yuan, T. Mai, M.G. Ma, Novel $Ti_3C_2T_x$ MXene wrapped wood sponges for fast cleanup of crude oil spills by outstanding joule heating and photothermal effect. J. Colloid Interface Sci. **606**, 971–982 (2022). https://doi.org/10.1016/j.jcis.2021.08.092

[S19] B. Zhou, J. Song, B. Wang, Y. Feng, C. Liu et al., Robust double-layered ANF/MXene-PEDOT:PWW Janus films with excellent multi-source driven heating and electromagnetic interference shielding properties. Nano Res. **15**(10), 9520–9530 (2022). https://doi.org/10.1007/s12274-022-4756-x

[S20] Q. Yuan, L.Z. Huang, P.L. Wang, T. Mai, M.G. Ma, Cellulose nanofiber/molybdenum disulfide aerogels for ultrahigh photothermal effect. J. Colloid Interface Sci. **624**, 70–78 (2022). https://doi.org/10.1016/j.jcis.2022.05.102

[S21] B. Zhou, G. Han, Z. Zhang, Z. Li, Y. Feng et al., Aramid nanofiber-derived carbon aerogel film with skin-core structure for high electromagnetic interference shielding and solar-thermal conversion. Carbon **184**, 562–570 (2021). https://doi.org/10.1016/j.carbon.2021.08.067

[S22] H. Liu, Z. Cui, L. Luo, Q. Liao, R. Xiong et al., Facile fabrication of flexible and ultrathin self-assembled $Ti_3C_2T_x$/bacterial cellulose composite films with multifunctional electromagnetic shielding and photothermal conversion performances. Chem. Eng. J. **454**, 140288 (2023). https://doi.org/10.1016/j.cej.2022.140288

[S23] J. Dong, S. Luo, S. Ning, G. Yang, D. Pan et al., MXene-coated wrinkled fabrics for stretchable and multifunctional electromagnetic interference shielding and electro/photo-thermal conversion applications. ACS Appl. Mater. Interfaces **13**(50), 60478–60488 (2021). https://doi.org/10.1021/acsami.1c19890
